# Effects of PLA-Based Antimicrobial Composite Films and Low-Energy X-Ray Irradiation on the Quality and Shelf Life of Strawberries

**DOI:** 10.3390/foods15162836

**Published:** 2026-08-14

**Authors:** Muhammed R. Sharaby, Stephane Salmieri, Elizabeth Ramirez Rodriguez, Lily Jaiswal, Monique Lacroix

**Affiliations:** 1 Armand-Frappier Health Biotechnology Research Centre, Institut National de la Recherche Scientifique (INRS), Research Laboratories in Sciences, Applied to Food (RESALA), Canadian Irradiation Centre (CIC), International Atomic Energy Agency (IAEA) Collaborating Centre, Institute of Nutrition and Functional Foods (INAF), Laval, QC H7V 1B7, Canada; muhammed.sharaby@inrs.ca (M.R.S.); stephane.salmieri@inrs.ca (S.S.); elizabeth.rodriguez@inrs.ca (E.R.R.); lily.jaiswal@ul.ie (L.J.); 2 Department of Botany and Microbiology, Faculty of Science, Alexandria University, Alexandria 21511, Egypt; 3 Department of Biological Sciences, Faculty of Science and Engineering, University of Limerick, V94 T9PX Limerick, Ireland

**Keywords:** antimicrobial, essential oils, low-energy X-ray, PLA, shelf life, strawberry

## Abstract

Strawberry is a nutritive fruit with a short life and postharvest losses. In this study, the quality and shelf life of strawberries during storage at 4 °C were evaluated after applying hurdle treatment of packaging with active polylactic acid (PLA)/Gelatine (G)-based films loaded with essential oils (EOs) and/or AgNPs and combined with low-energy (L-E) X-ray at a dose of 0.5 kGy. The results showed that the addition of EOs, AgNPs, or their combination decreased the tensile strength (TS) of films from 16.70 to 6.65–10.01 MPa and increased elongation at break (Eb) from 215.4 to 218.2–398.2%. The oxygen transmission rate (OTR) of films increased from 219.7 to 274.8–298.6 cm^3^/m^2^·day, which promoted the development of gas selective films for equilibrium-modified atmosphere packaging (EMAP). A level of 20% decay (primary shelf life criterion) of strawberries was observed on day 8 for the commercial control (polyethylene terephtalate clamshell), on day 9 for the PLA/G-AgNPs films, and on day 10 for the PLA/G-EOs films and PLA/G-EOs + AgNPs films. The combined application of the active films with L-E X-ray irradiation (PLA/G/EOs + AgNPs + I) extended the time to reach 20% decay to day 13 compared to day 9 for irradiated commercial control. An EMAP was achieved after 6–9 days of storage, with a gas composition around 13–14% O_2_ and 8–9% CO_2_. The combined treatment of EOs, AgNPs containing films, and L-E X-ray irradiation prevented firmness and total soluble solid loss of strawberries. The redness of strawberries and their total phenolic/anthocyanin content increased after irradiation treatment and maintained significantly higher levels (*p* ≤ 0.05) than those of non-irradiated samples during storage. This study demonstrates the effectiveness of combining active PLA/G films and L-E X-ray irradiation in extending the shelf life and preserving the quality of fresh strawberries.

## 1. Introduction

Strawberries (*Fragaria* × *ananassa*) are among the most important and widely consumed fruit crops worldwide and are included in the top five most popular fruits in Canada [1]. They are highly valued for their high content of nutritional and bioactive compounds such as anthocyanins, flavonoids, phenolic acids, and vitamin C [2]. Strawberries are non-climacteric fruits that need to be harvested fully ripe, and their delicate skin is prone to wounds and bruises that facilitate spoilage by *Rhizopus stolonifer*, one of the most common pathogens for strawberry [3,4] besides *Asperigillus niger* [5,6]. Previous studies have also found that the bacterial microbiota present on strawberries is complex, including potential plant pathogens, opportunistic pathogens, and human pathogens, such as *E. coli* O17:H7, *S. aureus*, and *Pseudomonas* spp. [7,8,9]. Strawberries are highly perishable fruits with a very short shelf life, especially in the face of temperature fluctuations and abusive storage [10]. Storage of strawberries at refrigeration temperatures in combination with modified atmosphere packaging (MAP) can delay their microbial spoilage and deterioration rate [11].

Conventionally, strawberries are packaged in single-use polyethylene terephthalate (PET) containers that fail to prevent spoilage, leading to both plastic pollution and food waste that can represent up to 40% of production [12,13]. Conventional packaging, such as clamshell containers, typically includes holes to reduce the relative humidity inside of the packaging due to the high water vapor barrier properties of PET, which increases the risk of water condensation, allowing fungal growth [14]. However, this strategy exposes the fruits to water loss, pathogen contamination, and atmospheric gases that accelerate spoilage of strawberries. On the contrary, storage of strawberries under MAP conditions with typically 5–10% O_2_ and 5–20% CO_2_ has shown efficacy in lowering the respiration rate and improving their shelf life [15].

Polylactic acid (PLA) is a biodegradable and renewable bioplastic that has gained popularity due to its thermoforming properties, biocompatibility, and versatility and the ability to enhance its physical properties through the incorporation of active ingredients [16]. Brittleness, low elongation at break (Eb), and poor thermal stability are the main drawbacks of PLA as a packaging film [17,18]. Blending with other polymers, such as proteins, can help improve the mechanical properties of PLA and reduce the cost of production [19]. For instance, Pizzoli, et al. [20] studied the effect of gelatin (G) in combination with PLA and TPS in extruded films and observed that G addition increased the water vapor permeability (WVP) and improved the surface morphology of the blend, a promising result for fruit packaging applications. High WVP is desirable for fruit packaging as it prevents water condensation, one of the main factors in fungal growth [14]. Essential oils (EOs), known for their antioxidant and antimicrobial activities, are generally recognized as safe (GRAS) agents that can be used for food preservation applications. Previous studies report that EOs are considered valuable ingredients for developing active packaging films that can be applied to improve the preservation and extend the shelf life of strawberries [21]. Metallic nanoparticles, especially silver nanoparticles (AgNPs), also demonstrate strong antimicrobial activity at a low concentration and have been widely applied to food packaging films [22]. Besides their strong inhibitory effect against microorganisms, NP-containing active films have shown improved thermomechanical, UV-protective, and oxygen/moisture barrier properties [23]. A previous study by Zhang et al. [24] showed that PLA-AgNP films effectively reduced total bacterial counts and weight loss of strawberries and delayed softness and soluble solid loss of strawberries during refrigerated storage without affecting sensory properties. However, the use of AgNPs in food packaging technologies still faces concerns regarding AgNPs’ potential impact on health and the environment, raising consumer safety issues upon the release of AgNPs from packaging into foods. Consequently, the European Food Safety Authority (EFSA) has set a permissible limit of Ag migration from packaging films to foods at 50 µg Ag/kg [25]. High migration above these limits can induce significant toxicological concerns caused mainly by cellular oxidative stress, reactive oxygen species (ROS) imbalance, and accumulation in vital organs [26]. So, some analyses are required to be performed, such as in vitro genotoxicity, absorption, metabolism, and excretion tests, before the approval of films’ application in the market [27]. However, previous studies have reported lower overall and specific migration of nano silver than the permissible limits of PLA-based films [22,28,29].

To better control postharvest losses of strawberries, treatments such as low-energy (L-E) X-ray irradiation can be applied along with active packaging. Irradiation is a non-thermal technique that offers the ability to process packaged food within a short period of time [30,31]. Compared to other sources of irradiation, L-E X-ray has high linear energy transfer (LET) and a high relative biological effect (RBE) that is efficient against pathogens; it also has low requirements for a protection shield, low costs, and a compact size, which make it a promising technique for food applications [32,33]. Most of the studies on strawberry irradiation have focused on gamma irradiation, electron beams, and high-energy X-ray, demonstrating the potential of these technologies in extending the shelf life of strawberries [30,34,35]. However, research exploring the application of L-E X-ray irradiation remains new, presenting an opportunity to further investigate its effect on postharvest strawberry preservation.

Therefore, this study aimed to investigate the in situ beneficial effect of PLA/G-based bioactive films containing EOs and AgNPs in combination with L-E X-ray irradiation to preserve the quality of strawberries and extend their shelf life. The shelf life and physicochemical and nutritional properties of strawberries were evaluated along with the gas composition inside of the package during 27 days of storage time at 4 ± 1 °C.

## 2. Materials and Methods

### 2.1. Materials

Amorphous PLA Luminy^®^ LX975 (M_w_ 60,000 g/mol, density 1.24 g/ cm^3^, 88% L-isomer purity, melting temperature 130 °C, glass transition temperature of 60 °C) was purchased from Shanghai Terppon Chemical Co., Ltd. (Shanghai, China). Glycerol, m-Phosphoric acid, potassium phosphate monobasic, potassium phosphate dibasic, DTT (dithiothreitol), sodium triacetate trihydrate, and potassium hydroxide were obtained from Thermo Fisher Scientific (Saint-Laurent, QC, Canada). Gelatin, gallic acid, Folin–Ciocalteu reagent, potassium chloride, hydrochloric acid, sodium acetate anhydrous, phosphoric acid, and calcium chloride were acquired from MilliporeSigma Canada Ltd. (Oakville, ON, Canada). Savory thyme (S. thyme), Thyme ct thymol (T. thymol), and Cinnamon cassia (C. cassia) EOs were supplied by Zayat Aroma (Bromont, QC, Canada). AgNPs were provided by NanoBrand (Laval, QC, Canada). These AgNPs were predominantly spherical, with an average diameter of 64 nm and a low polydispersity index (0.18 ± 0.01). Potato dextrose broth (PDB), potato dextrose agar (PDA), tryptic soy broth (TSB), and tryptic soy agar (TSA) were obtained from Alpha Biosciences (Baltimore, MD, USA). Strawberries were purchased from a local supermarket (Laval, QC, Canada).

### 2.2. Preparation of PLA/G Films

Prior to processing, PLA pellets and G powder were dried at 65 °C overnight to eliminate residual moisture. The composite films were prepared using PLA, G (80:20 *w*/*w*), glycerol 20% (*w*/*w* of polymers), loaded with 7.5% (*w*/*w* of polymers) of an EO blend (S. thyme, T. thymol, and C. cassia at a ratio of 1:2:6 *v*/*v* determined based on preliminary studies, and 100 ppm of AgNPs. The components were melt-compounded in an Xplore twin-screw micro compounder MC 15 HT (Nanoscience Instruments, Alexandria, VA, USA) at 130 °C, 100 rpm for 2 min. The film formulations were hot-pressed using a Carver hydraulic press unit (model 3912; Carver Inc., Wabash, IN, USA) at 120 °C for 2 min under 4 tons of pressure to prepare thin films that were used in the packaging of strawberries and for further characterization. Four (4) groups were prepared: film without antimicrobial compound used as control (PLA/G), film with EOs (PLA/G-EOs), film with AgNPs (PLA/G-AgNPs), and film with EOs + AgNPs blend (PLA/G-EOs + AgNPs).

### 2.3. Film Characterization

#### 2.3.1. Water Solubility

The water solubility (WS) of the films was tested at room temperature according to the method of Sharaby et al. [36]. Films were cut at approximately 2 cm^2^ and subsequently dried at 50 °C until a constant weight was achieved (*W*_1_). The dried films were placed in 100 mL of water at room temperature and left to soak for 24 h. The films were removed from the water and set to dry in the previous set conditions (*W*_2_). The final weight for each treatment was then determined and expressed as a percentage (%). The water solubility was calculated using Equation (1):

*WS* (%) = (*W*_1_ − *W*_2_)/*W*_1_ × 100 (1)

#### 2.3.2. Physical Appearance and Mechanical Properties

The physical appearance of the films was visually evaluated. The thickness, tensile strength (TS), Young’s modulus (YM), and elongation at break (Eb) of the 4 different groups of films were evaluated following the ASTM D638-99 method [37]. Thickness was measured using a Mitutoyo Digimatic Indicator 543-792B (model ID-S112EX; resolution: 1 μm; BDI Canada Inc., Laval, QC, Canada) at 5 random positions around the film. Film width was measured using a Traceable Carbon Fiber Digital Caliper (resolution: 0.1 mm; accuracy: ±0.2 mm; Thermo Fisher Scientific). Films were cut into 60 × 10 mm rectangular strips, and the TS (MPa), YM (MPa), and Eb (%) of each group were evaluated with a Universal Testing Machine (UTM) H5KT (Tinius Olsen Testing Machine Co., Inc., Horsham, PA, USA) and using Horizon software ver. 10.3.1.10. The initial grip separation was set at 30 mm, crosshead speed of 50 mm/min, and load cell of 100 N. Tests were performed in 6 replicates/formulation.

#### 2.3.3. Water Vapor Permeability (WVP)

The water vapor permeability (WVP) was determined gravimetrically using the ASTM E96/E96M-16 procedure [38]. Film thickness was measured using a Mitutoyo Digimatic Indicator 543-792B (model ID-S112EX; BDI Canada Inc.) at 5 random positions around the film. They were then sealed onto Vapometer E-Z cups (Model No. 68-3000, Twhing-Albert Instrument Co., West Berlin, NJ, USA), each containing 30 g of anhydrous calcium chloride (0% RH). After initial weighing, the cells were placed in a Shellab 9010 L controlled humidity chamber (Sheldon Manufacturing Inc., Cornelius, OR, USA) set at 25 °C and 50 ± 1% RH for 24 h. The difference in weight corresponds to the water vapor absorbed by the desiccant. The WVP was determined following Equation (2):

*WVP* = (Δ*w* × *x*)/(*A* × Δ*P*) (2)

where (Δ*w*) is the weight gain of the cell (g) after 24 h, (*x*) is the film thickness (mm), (*A*) is the area of exposed film (31.67 × 10^−4^ m^2^), and (Δ*P*) is the differential vapor pressure of water through the film (Δ*P* = 3.2823 kPa at 25 °C/60% RH).

#### 2.3.4. Oxygen Transmission Rate (OTR)

The oxygen transmission rate (OTR) of films was determined using the ASTM D-3985 procedure with some modifications [39]. Film thickness was measured using a Mitutoyo Digimatic Indicator 543-792B (model ID-S112EX; BDI Canada Inc.). OTR was evaluated utilizing an OX-TRAN^®^ 1/50 (AMETEK MOCON Inc., Brooklyn Park, MN, USA). Tests were performed at 23 ± 1 °C under atmospheric conditions. The film area was 5 cm^2^. Tests were carried out in continuous test mode until curve equilibrium. The OTR of films was expressed in cm^3^/m^2^∙day.

#### 2.3.5. Water Contact Angle (WCA)

The surface wettability of the prepared best films was evaluated based on water contact angle (WCA) measurements using an optical tensiometer (Biolin Scientific, Espoo, Finland) operated in sessile drop mode. The instrument was equipped with a high-resolution digital camera and accompanying image analysis software for droplet shape evaluation. A deionized water droplet (10 μL) was gently deposited onto the surface of each film using a microsyringe under ambient conditions (T: 23 ± 0.5 °C). The contact angle was recorded immediately after droplet deposition to minimize the effects of evaporation and liquid absorption. To ensure reproducibility and account for surface heterogeneity, measurements were performed at five randomly selected positions on each film surface. Each contact angle value corresponded to the mean value of the left and right contact angle at a given point in time. Three independent specimens were analyzed for each film, and the final values were reported as mean ± standard deviation.

#### 2.3.6. Fourier Transform Infrared (FTIR) Spectroscopy

Fourier transform infrared (FTIR) spectra of the films were recorded using a Spectrum One spectrophotometer (PerkinElmer, Woodbridge, ON, Canada) equipped with an attenuated total reflectance (ATR) device for analysis of solids and a high linearity lithium tantalate (HLLT) detector. Film specimens were pre-conditioned at 25 °C/57.5% relative humidity (RH) for 48 h in a desiccator containing a saturated solution of sodium bromide. Spectra were analyzed by using Spectrum^®^ 10.7.2.1630 software. Films were placed onto a zinc selenide crystal, and the analysis was performed after the background within the spectral region of 650–4000 cm^−1^ with 64 scans recorded at a 4 cm^−1^ resolution. After attenuation of total reflectance and baseline correction, spectra were normalized within the whole infrared region with the minimum transmission peak set at 75%. Molecular interactions between active compounds and the polymeric matrix were assessed via the identification of vibration peaks. Tests were performed at 23 °C/50% RH.

#### 2.3.7. Thermogravimetric Analysis (TGA)

The influence of the addition of EOs and AgNPs on the thermal stability of the bioactive films was analyzed through thermogravimetric analysis (TGA) according to Jaiswal et al. [39]. Film samples (10–20 mg) were heated from 30 to 600 °C at 10 °C/min under a nitrogen atmosphere using a thermogravimetric analyzer TGA 4000 (PerkinElmer). The weight loss (%) of the samples was determined from the TGA curves. Derivative thermogravimetric (DTG) curves were obtained using the first derivative of the TGA curves and allowed for determination of the maximum decomposition temperature (T_max_) of the samples.

#### 2.3.8. Differential Scanning Calorimetry (DSC)

DSC analysis was performed using a DSC 4000 calorimeter (PerkinElmer) to investigate the glass transition of films. Pre-conditioned film samples (5–10 mg) were sealed hermetically in a 40 µL standard aluminum pan (PerkinElmer) and subjected to a heat–cool–heat cycle process under a nitrogen flow rate of 20 mL/min. Firstly, samples were heated from 30 °C to 200 °C at a rate of 10 °C/min. Then, samples were cooled from 200 to 30 °C at 5 °C/min. In the end, the 2nd heating cycle was performed up to 200 °C at a rate of 10 °C/min. The glass transition temperature (Tg), taken at the mid-point of heat capacity changes, was obtained from the first heating [40].

#### 2.3.9. Antimicrobial Activity

The fungal cultures (*R. stolonifer* ATCC 14038 and *A. niger* ATCC 1015) were stored at −80 ± 1 °C in PDB containing glycerol (10% *v*/*v*), and bacterial cultures (*E. coli* O157:H7 ATCC 35150 and *S. aureus* ATCC 29213) were stored at −80 ± 1 °C in TSB containing glycerol (10% *v*/*v*). Prior to the experiment, the cultures were passed through two consecutive cycles in TSB for 24 h at 37 °C and in PDB for 48 h at 28 °C. Bacterial cultures were centrifuged and washed with saline water to obtain the bacterial concentration to 10^5^ CFU/mL for each experiment. Fungal cultures’ concentration was adjusted to 10^5^ spores/mL by collecting spores using saline water and filtered through a sterile screen (40 µm nylon screen).

The antimicrobial activity of the bioactive packaging films containing EOs and AgNPs was determined in vitro using an agar diffusion assay [41]. Film samples (1 cm × 1 cm, 400 mg) were placed in the middle of a previously inoculated TSA and PDA plate using 0.1 mL of each bacterial (10^5^ CFU/mL) and spore suspension (10^5^ spores/mL). All plates were the incubated at 28 ± 1 °C for 48 h. Results were expressed in inhibitory capacity (*IC*%) calculated according to Equation (3):

*IC* = *D_IZ_*/*D_PD_* × 100 (3)

where *D_IZ_* is the diameter of the inhibition zone (no colony growth) and *D_PD_* is the diameter of the Petri dish.

### 2.4. Application of the Bioactive Films in Strawberry Preservation

#### 2.4.1. Experimental Design

Ten (10) groups of strawberry packaging were evaluated during storage as follows: commercial control: non-modified PET clamshell; Group 1: PLA/G control film without antifungal compounds; Group 2: PLA/G-AgNPs film; Group 3: PLA/G-EOs film; and Group 4: PLA/G-EOs + AgNPs film. The samples that were X-ray irradiated were designated as commercial control + I: irradiated non-modified PET clamshell; Group 1 + I: irradiated PLA/G control film without antifungal compounds; Group 2 + I: irradiated PLA/G-AgNPs film; Group 3 + I: irradiated PLA/G-EOs film; and Group 4 + I: irradiated PLA/G-EOs + AgNPs film. The independent factors included the addition of a bioactive compound (AgNPs and EOs) and the treatment with/without irradiation.

#### 2.4.2. Fruit Samples

Strawberries were selected based on size uniformity, color, and lack of visual defect or microbial infection. The fruits were washed with 1% (*v*/*v*) sodium hypochlorite for 1 min and then rinsed with distilled water and subsequently dried at room temperature for 30 min. Following preparation, 10 fruits were randomly distributed in each packaging treatment and stored at 4 ± 1 °C.

#### 2.4.3. Design of Modified Packaging

**Figure 1:**
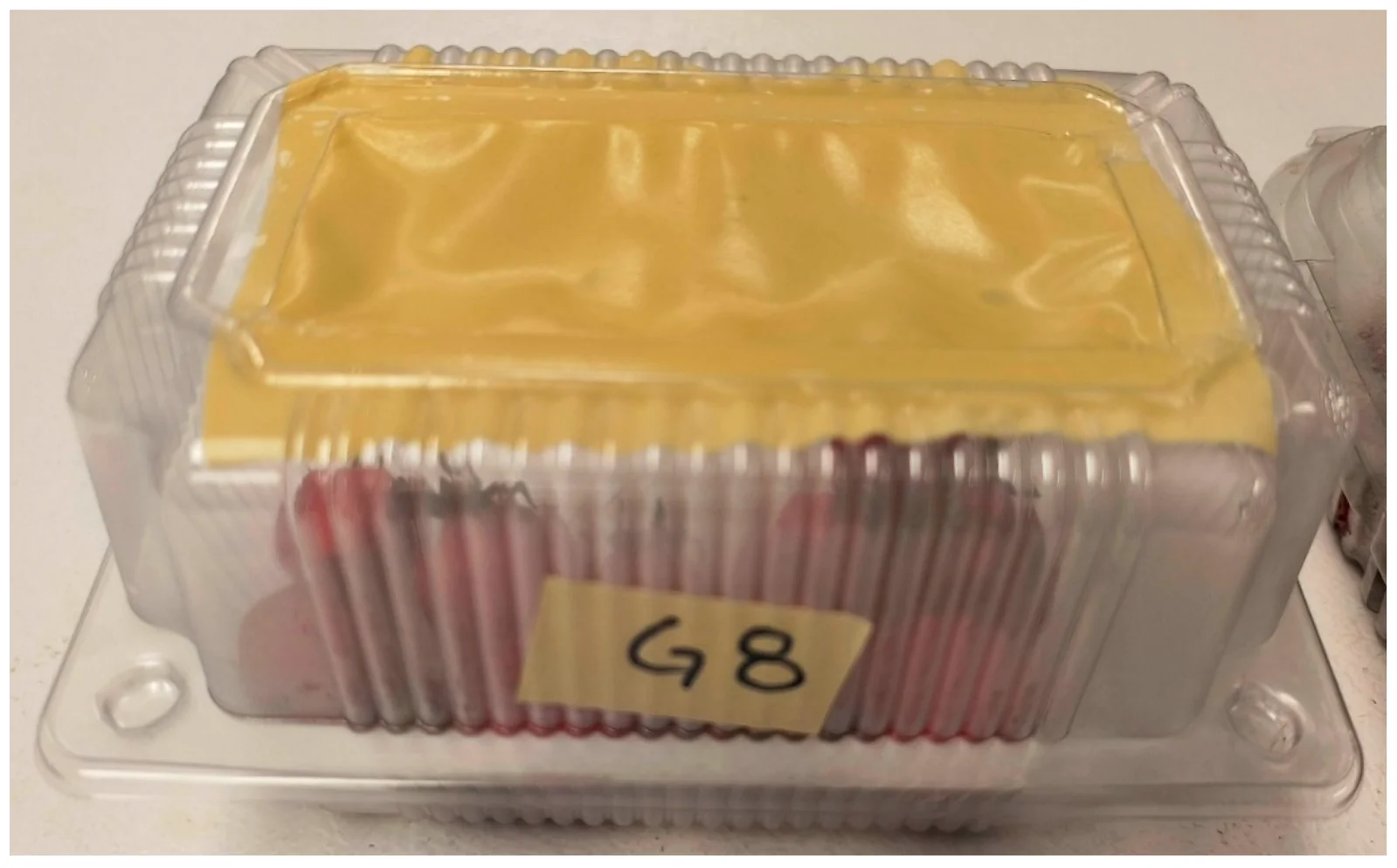
Design of active packaging system containing strawberries. The active film (PBAT/G containing active compounds) was hermetically sealed onto the PET clamshell cover.

PET clamshells were used as storage containers. The lid of each package was modified by cutting a window of 4.5 × 11.5 cm. The window was hermetically sealed (using double-sided hermetic tape and silicon glue) with the different packaging biopolymer films, as illustrated in Figure 1.

#### 2.4.4. X-Ray Irradiation

PET clamshells containing 10 strawberries were irradiated at 0.5 kGy using a MultiRad 350 X-ray irradiator (Precision X-ray, North Branford, CT, USA) at a dose rate of 1.5 kGy/h for 20 min operating at 55 kV and 29.8 mA in Auto-Dose Control mode, without filter, and on a turntable positioned at 29.5 cm from the source. Dosimetry measurements were performed using GEX B3 dosimeter films (GEX Corp., Palm City, FL, USA), and doses of irradiation were estimated through measurements at 552 nm. Dosimetry measurements were also compared with data obtained from the internal Varex Claymount G1002 ion chamber integrated into the MultiRad 350 X-ray irradiator.

### 2.5. Determination of Strawberry Quality

Parameters such as decay rate, weight loss, headspace gas composition, color, firmness, total soluble solids (TSS), pH, total phenolic content (TPC), anthocyanin, and vitamin C content were evaluated during storage at days 0, 6, 9, 12, 18, and 24.

#### 2.5.1. Decay Percentage

Strawberries with visible mold infection, brown and white spots, or softening were considered decayed. The results were reported as a percentage of decay according to Equation (4) [29]:

(4) $$Decay\;percentage\;\left(\%\right)=\;\frac{Number\;of\;decayed\;samples}{Total\;number\;of\;samples}\times 100$$

#### 2.5.2. Weight Loss

The cumulative weight loss percentage of the total fruits in the package was calculated according to Khalil et al. [42] following Equation (5):

(5) $$Weight\;loss\;(\%)=\;\frac{Wi-Wt}{Wi}\;\times 100\%$$

where *Wi* = initial weight of the fruits and *Wt* = weight of the fruits on day *t*.

#### 2.5.3. Headspace Gas Composition

The gas composition inside the packaging was assessed using a Systech 6600 Headspace O_2_/CO_2_ analyzer (Industrial Physics, New Albany, IL, USA). Results were expressed as percentages (%) of O_2_ and CO_2_. To prevent gas leakage, a plastic septum (Meyer Service & Supply Ltd., Long Sault, ON, Canada) was previously installed on the package to facilitate gas sampling inside of the package. Sampling was performed using a 21 G × 1 in. needle (BD^®^ Precision Glide™ needle; Becton Dickinson and Co., Mississauga, ON, Canada) and a 0.45 μm syringe filter (Sarstedt Inc., Montreal, QC, Canada) onto the probe.

#### 2.5.4. Surface Color

The color of the strawberries was evaluated with a color reader CR-10 Plus (Folio Instruments Inc., Kitchener, ON, Canada) by using the CIELAB color scale. Six (6) measurements were taken per strawberry sample in the equatorial section of the strawberries to determine the values of lightness (L*), redness (a*), and yellowness (b*).

#### 2.5.5. Firmness

The firmness (resistance of penetration) of strawberries was measured using a Stevens-LFRA TA-1000 texture analyzer (Texture Technologies Corp., Scarsdale, NY, USA). Penetration tests were conducted with a 2 mm diameter flat head cylindrical probe that penetrated 5 mm into each strawberry at a speed of 1 mm/s [21]. Firmness was expressed in Newton (N).

#### 2.5.6. Biochemical Determinations

Three (3) strawberries from each treatment were blended and centrifuged at 5500× *g* for 20 min at 4 °C. The supernatant was filtered using a medium-speed filter under vacuum. The samples were stored closed tightly at 4 °C in the dark until further biochemical analyses [43].

##### Total Soluble Solids (TSS) and pH

Total soluble solids (TSS) from the strawberry puree were measured using an RHB-32ATC refractometer (Thermo Fisher Scientific) and expressed in °Brix. The pH of the strawberry puree was determined with a digital accumet AB15 pH meter equipped with an aqueous Ag/AgCl reference electrode (Thermo Fisher Scientific).

##### Total Phenolic Content (TPC)

The total phenolic content (TPC) in the strawberry puree was determined using the Folin–Ciocalteu reagent [44]. After reaction, the absorbance of the samples was measured at 760 nm, using a Cary 8454 UV-visible spectrophotometer (Agilent Technologies Canada Inc., Saint-Laurent, QC, Canada). A standard curve with gallic acid was established, and results were expressed in mg of gallic acid equivalents (GAE)/100 g of fruit.

##### Anthocyanin Content

The total monomeric anthocyanin pigment concentration was evaluated using the pH differential method according to Sharaby et al. [36]. The pH 1.0 buffer was prepared with potassium chloride 0.025 M and HCl. The pH 4 buffer was prepared using sodium acetate 0.4 M and HCl. A sample of 2.5 mL of strawberry puree was diluted with each buffer to a final volume of 25 mL. The absorbance was measured at 520 and 700 nm using a UV-visible Scinco S-3100 UV-visible spectrophotometer (Scinco Co., Ltd., Seoul, Republic of Korea). The anthocyanin pigment concentration was calculated using Equation (6) and expressed as mg of cyanidin-3-glucoside equivalents (CGE)/L of strawberry puree.

(6) $$Anthocyanin\;pigment=\frac{A\times Mw\times DF\times 10^{3}}{\varepsilon }$$

$$A=\left(A_{520\mathrm{nm}\;}-A_{700\mathrm{nm}}\right)pH\;1.0-\left(A_{520\mathrm{nm}\;}-A_{700\mathrm{nm}}\right)pH\;4.5$$

where *Mw* = 449.2 g/mol for cyanidin-3-glucoside; *DF* is the dilution factor; and *ε* is the molar absorption coefficient (=26,900 L/mol·cm).

##### Vitamin C Content

The determination of ascorbic acid (AA) and dehydroascorbic acid (DHA) was performed using the kit VitaFast^®^ Vitamin C P1010 (R-Biopharm AG, Darmstadt, Germany) according to the instructions of the manufacturer using a 96-well microplate. The absorbance was read at 562 nm utilizing a microplate reader ELx800 (BioTek Instruments, Inc., Winooski, VT, USA). Vitamin C content was determined according to Equation (7) and expressed as mg of total ascorbic acid/100 g of fruit, mg of ascorbic acid/100 g of fruit, and mg of dehydroascorbic acid/100 g of fruit.

(7) Dehydroascorbic acid=Total ascorbic acid−Free ascorbic acid

### 2.6. Statistical Analysis

For all experiments, measurements were performed in triplicate (n = 3), with three analyses performed for each replicate. The results are expressed as mean values with their corresponding standard deviations (mean ± SD). One-way ANOVA and Duncan’s multiple range test were used to evaluate significant differences among means using IBM SPSS Statistics software (version 28.0; IBM Canada Ltd., Markham, ON, Canada). The *p*-value was considered statistically significant when *p* ≤ 0.05.

## 3. Results and Discussion

### 3.1. Biopolymer Packaging Film Properties

#### 3.1.1. Water Solubility

The WS values of the films are displayed in Table 1. The results show that the films had a low WS ranging from 1.15 to 1.59%. Generally, a low WS is advantageous for packaging high moisture produce like strawberries, especially in highly humid environments, as it allows for a reduction of surface water activity and microbial contamination risk during storage and handling [45]. The addition of AgNPs had no significant effect (*p* > 0.05) on film solubility. The WS increased significantly (*p* ≤ 0.05) in the presence of EOs by around 33% compared to the PLA/G control (from 1.18 to 1.59%). However, this difference is very slight and does not compromise the application of such films. This increase may be attributed to the disruption of the blend matrix, easing the water’s access to the polymeric matrix, resulting in more solubility of gelatine in the blend film [46]. Although EOs are hydrophobic compounds, it was observed in previous studies that their migration from PLA into water increased the water solubility of the biopolymer [47].

#### 3.1.2. Films’ Appearance and Mechanical Properties

As shown in Figure S1, all of the PLA/G-based films were macroscopically homogenous and free-standing, with smooth surfaces devoid of visible cracks, surface deformations, or holes. The PLA/G control film appeared white and semi-translucent. In contrast, the PLA/G-AgNPs had a light brownish color caused by the AgNPs [48], with no visible agglomerates in the film. The PLA/G-EOs film had more opaque and yellowish appearance with even distribution across the film due to the intrinsic color of the EO components. A deeper yellow color evolved in the PLA/G-EOs + AgNPs, with high opacity.

Mechanical characterization of films is important for food packaging applications to ensure films’ ability to tolerate external stress. The results of TS (MPa), YM (MPa), and Eb (%) of the films are displayed in Table 1. There was no significant difference (*p* > 0.05) in the thickness between the different groups with the incorporation of antimicrobial compounds (ranging from 122.2 to 149.4 µm). The TS of the control PLA/G film (16.70 MPa) decreased significantly (*p* ≤ 0.05) with the incorporation of AgNPs, EOs, and their combination by 40% (10.01 MPa), 53% (7.78 MPa), and 60% (6.65 MPa), respectively. Similarly, YM also decreased significantly (*p* ≤ 0.05) by, respectively, 42% (292.8 MPa), 77% (116.0 MPa), and 94% (32.6 MPa) with the addition of AgNPs, EOs, and their combination compared to the control (503.4 MPa). These results suggest a more elastic behavior of the material with the incorporation of active compounds. An opposite trend was observed with Eb, where the films showed significant enhanced stretchability (*p* ≤ 0.05) with the incorporation of EOs (385.8–389.2%) compared to the control (215.4%). EO addition could decrease the molecular interactions between PLA chains, decreasing the TS values and increasing the Eb because of their plasticizing effect [49]. Ramos et al. [50] found decreased TS and YM and increased Eb in a PLA-AgNPs–thymol composite film, in line with this study. The authors suggested that presence of AgNPs helped to increase thymol mobility, which was reflected in the increased dispersion of the AgNPs in the PLA matrix; these actions were enhanced by the intermolecular interactions between thymol and the partial positive charge on the surface of the AgNPs.

#### 3.1.3. WVP

Barrier properties against water vapor play a key role in strawberry storage, as they can induce water vapor condensation inside of the packaging, increasing the risk of fungal growth. The effect of AgNPs and EOs on the water vapor permeability (WVP) of films is summarized in Table 1. The WVP values ranged from 1.74 (PLA/G film) to 1.93 (PLA/G-EOs + AgNPs) g·mm/m^2^·day·kPa. The inclusion of AgNPs was previously reported to improve the barrier properties of nanocomposite films by creating tortuous pathways for moisture diffusion. This observed trend suggests that AgNPs in the films have affected the continuity of the blended matrix, created voids, and formed free volume at the polymer interface [51], which resulted in increased WVP (1.90 g·mm/m^2^·day·kPaFurthermore, because EOs are hydrophobic compounds, their interactions can lead to a decrease in the WVP of PLA [52]. However, the plasticizing effect of EOs on the polymer matrix and the increase in the mobility of polymer chains, supported by the Eb% results (Table 1), as well as the strengthening of the interfacial bond between EOs and polymer, would hinder interactions between polymer chains and water molecules, leading to an increase in WVP [53]. The results obtained in this study indicate that the high WVP could be advantageous for hermetically sealed strawberry packages, as they compensate for the absence of perforations typically found in conventional clamshells. Meanwhile, insufficient WVP allows for moisture to accumulate and condense within the package, a main factor in fungal growth [14]. Excessive permeability may promote excessive moisture loss from the fruit, leading to weight loss and firmness deterioration, indicating that an optimal WVP range is required to balance moisture condensation control versus fruit dehydration.

#### 3.1.4. OTR

The effect of AgNPs and EOs on the OTR of films is summarized in Table 1. The control PLA/G film had an OTR value of 219.8 cm^3^/m^2^·day. Upon the incorporation of active compounds, the OTR significantly increased (*p* ≤ 0.05) by 25 to 36% (274.9–298.7 cm^3^/m^2^·day), with no significant differences (*p* > 0.05) observed between the active films. Notably, this trend aligns with the increase recorded in WVP across the same films, suggesting that both oxygen and water vapor transport are controlled by a common structural mechanism. In this context, EOs as hydrophobic compounds facilitated the diffusion of oxygen through the matrix, therefore increasing the OTR up to 274.89 cm^3^/m^2^·day [54]. The increase in OTR observed in PLA/G-AgNPs (from 219.8 to 290.03 cm^3^/m^2^·day) is consistent with a prior report that silver nanoparticles can compromise the gas barrier performance of polymer films [55], attributed to NP-induced disruption of the matrix and the generation of interfacial defects.

Caner et al. [56] demonstrated that the OTR of the lidding film is a decisive parameter for equilibrium MAP development in strawberry packaging. The authors observed that cast polypropylene (PP; OTR 1607 cm^3^/m^2^·day) preserved fruit quality more effectively than PET/ethylene vinyl alcohol/polyethylene–low-acetyl fractions (PET/EVOH-LAF; OTR 1.5 cm^3^/m^2^·day), which induced anaerobic respiration. In contrast, low-density polyethylene (LDPE; 4050 cm^3^/m^2^·day) led to accelerated deterioration due to fruit softening. Similarly, it was observed that O_2_ permeation of 0.5 mL/day·atm or lower led to anaerobic respiration and anoxia of strawberries [57]. When film permeability is insufficient relative to the respiration demand of strawberry, O_2_ depletion and packaging film of insufficient permeability can result in the development of undesirable fermentation reactions causing off-flavors and odors. Conversely, O_2_ availability supported by higher-OTR films helps sustain aerobic respiration, firmer fruits, reduced fungal decay, and improved flavor retention [58].

#### 3.1.5. Water Contact Angle (WCA)

Evaluating the films’ wettability is a vital surface characterization technique in the development of biodegradable active packaging films. The wettability of the control and PLA/G active films was evaluated, and the contact angle values are presented in Table 1. The control PLA/G film had WCA value of 69.66°, indicating hydrophilic nature of the film’s surface. The incorporation of AgNPs led to a slight reduction in WCA (*p* ≤ 0.05), indicating increased hydrophilicity of the film, in line with the results reported by Szymanska-Chargot et al. [59]. This may be attributed to the introduction of polar groups onto the surface of the films [60]. On the contrary, the PLA/G-EOs showed the highest WCA value (82.45°), attributed to the hydrophobic nature of the oils’ constituents, which insignificantly decreased (*p* > 0.05) to 81.35°. Previous studies have found similar results of increased surface hydrophobicity of the films upon incorporation of EOs [61,62].

#### 3.1.6. FTIR Analysis

**Figure 2:**
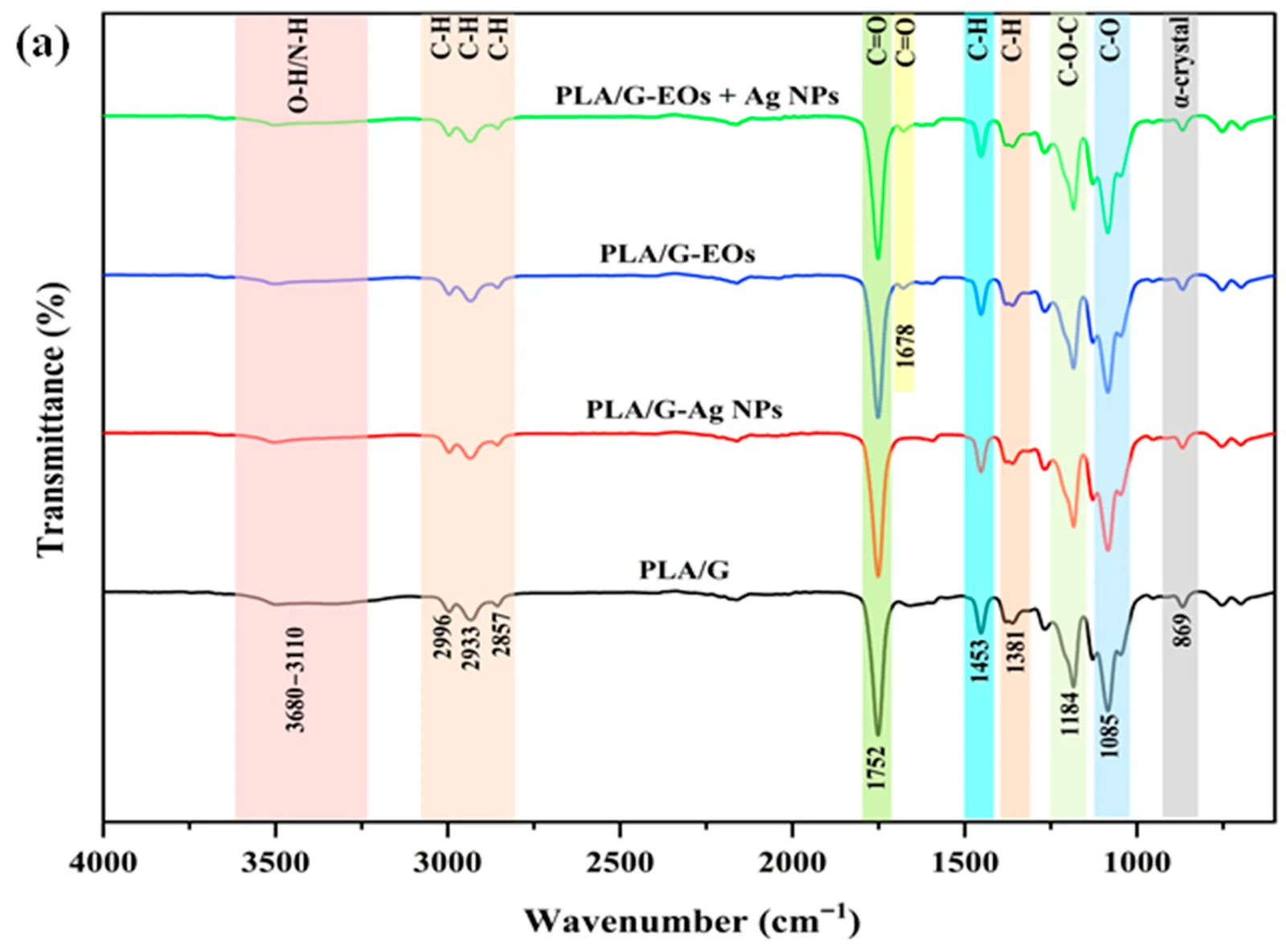

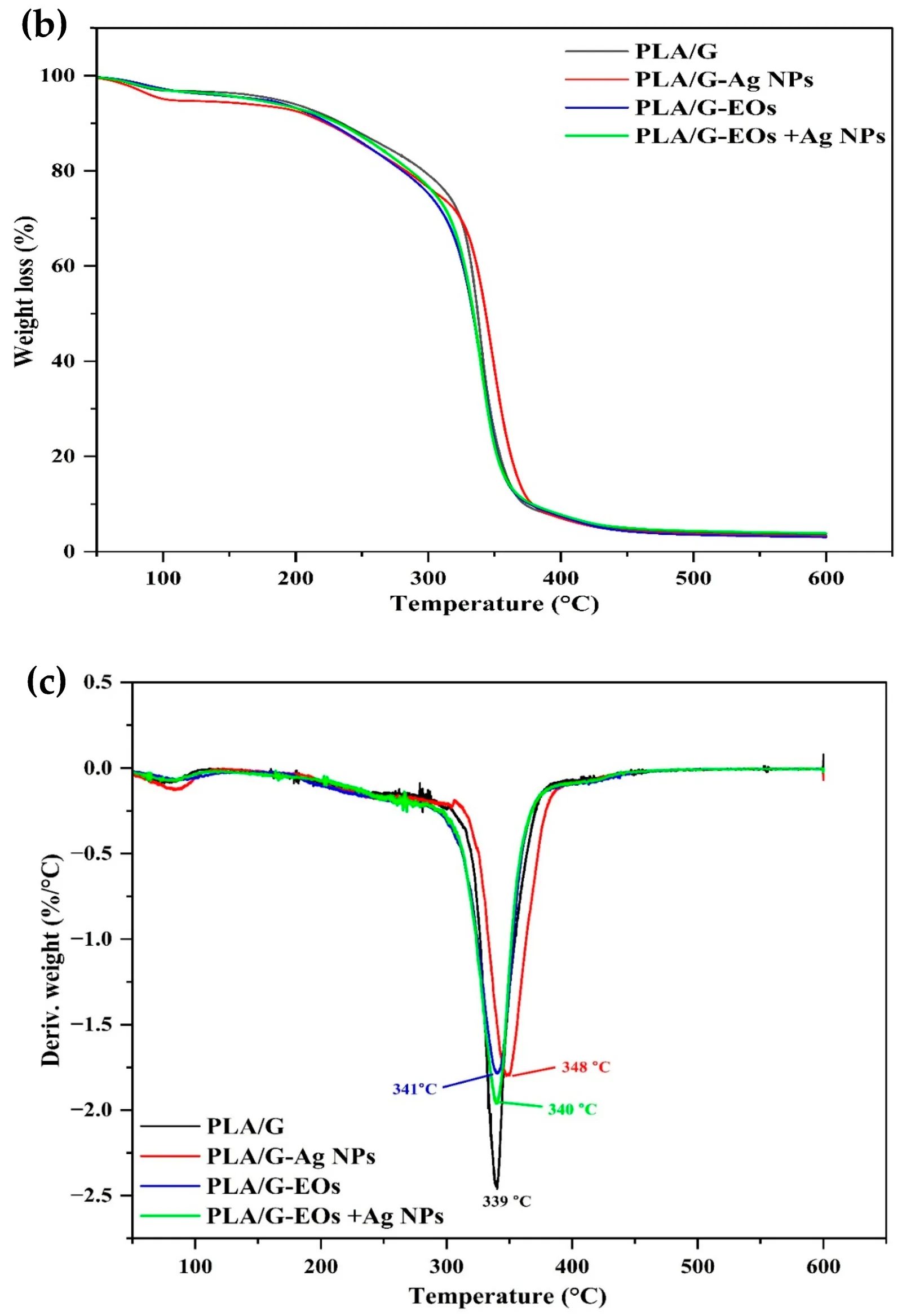
FTIR spectra (**a**), TGA (**b**), and DTG (**c**) curves of the PLA/G active packaging films.

FTIR analysis of the films was performed to investigate the intermolecular interactions between the polymer matrix and EOs/AgNPs. FTIR spectra are presented in Figure 2a. There was no evidence of molecular or chemical structure changes after the addition of EOs and AgNPs. All films displayed a broad band between 3680 and 3110 cm^−1^ corresponding to O–H stretching vibration, including N-H stretching vibration from the Amide A band of gelatin [39,63]. The peaks at 2996, 2933, and 2857 cm^−1^ are assigned to –CH stretching vibrations [64], and the strong peak located at 1752 cm^−1^ is mainly assignable to the ester C=O stretching of PLA. Peaks at 1453 cm^−1^ and 1381 cm^−1^ (C–H deformation), 1184 cm^−1^ (C–O–C stretching), 1085 cm^−1^ (C–O stretching), and 869 cm^−1^ (α-crystals relatively to amorphous composition) correspond to the characteristic peaks of PLA [47,65,66]. A small peak was observed at 1678 cm^−1^ in PLA/G-EOs and PLA/G-EOs +AgNPs films assigned to the aldehyde group (C=O) present in EOs [67,68]. This peak results in a shift towards lower frequencies from the ester C=O stretching of PLA. The absence of new peaks suggests that EOs did not bond chemically with the PLA/G films, which agrees with Gonon et al. [54], who reported that carvacrol and cinnamaldehyde, prevalent constituents in EOs, interact with PLA through van der Waals forces rather than covalent bonding. In the same context, no changes were observed in the FTIR spectra of the films by adding AgNPs, indicating no covalent interaction between PLA and AgNPs [22].

#### 3.1.7. TGA

The effect of EOs and AgNPs on the thermal stability of the film formulation was studied using TGA, as depicted in Figure 2b,c. All films showed similar thermal degradative behavior with an initial small mass loss and one major degradation stage (Figure 2b). The initial degradation stage ranged from 50 to 115 °C in all the films and corresponds to the loss of free and bound water and decomposition of possible hydrogen bonds in the films [69], resulting in a mass loss between 3.5 and 5.2% in all films. The main degradation stage ranged between 280 to 400 °C relative to the decomposition of PLA, G, and glycerol [70]. The maximum decomposition temperature (T_max_) of the PLA/G film without antimicrobial compounds was 339 °C. Incorporation of the EOs did not affect the thermal stability of the films (PLA/G-EOs, PLA/G-EOs +AgNPs), recording a T_max_ around 340 °C, in accordance with the results of Qin et al. [45]. On the other hand, the T_max_ value increased to around 348 °C in the PLA/G-AgNPs film (Figure 2c). This may be due to the thermal stability of the AgNPs that acted as a thermal barrier in early stages of thermal decomposition [70]. Liu et al. [71] reported a proportional increase in the thermal stability of PLA electrospun films loaded with AgNPs, observing that the T_max_ of PLA increased from 360 to 364 by adding 7% AgNPs. Additionally, it was previously suggested that AgNPs enhanced the thermal stability of PLA electrospun films by reducing the mobility of molecular chains of PLA and consequently slowing the degradation process, causing decomposition to occur at higher temperatures [69]. Furthermore, AgNPs can be considered to act as nucleating agents that enhance the crystallinity degree and hence increase the melting temperature of PLA.

#### 3.1.8. DSC

The DSC curves during the first heating scan of the prepared films are shown in Figure S2. The thermograms of the control and active PLA/G films revealed a single broad endothermic transition between 35 and 85 °C related to the Tg of the PLA/G blend and the melting of gelatine. This was also associated with the adsorbed water loss from the films [72,73]. The Tg of the control PLA/G film was 47.8 °C. The deceased Tg value of the film compared to the Tg of PLA alone in previous studies may be due to the plasticized nature of the film [40]. The active films had lower Tg values of 40.1, 44.3, and 42 °C for the PLA/G-AgNPs, PLA/G-EOs, and PLA/G-EOs + AgNPs films, respectively, suggesting the disruption of intermolecular interactions and enhancement of chain mobility, thereby lowering the required energy for segmental relaxation [50,74]. There was an absence of the crystallization and melting peaks in the thermograms of the films, which may be indicating that the PLA phase remained largely amorphous regardless of the incorporation of AgNPs, EOs, or their combination. This observation was similar to the findings of Zhao et al. [75] for the PLA/gelatine films prepared using the solvent casting method.

#### 3.1.9. Antimicrobial Activity

To evaluate the antimicrobial activity of the films with EOs and AgNPs, zone of inhibition assays was performed against two fungal and two bacterial strains. The results of the inhibitory capacity (IC, %) of the films are shown in Table 2. The control films did not exhibit any activity against any of the tested organisms (IC = 0%). The IC values for the PLA/G-AgNPs, PLA/G-EOs, and PLA/G-EOs + AgNPs films ranged between 18.79–29.80%, 23.15–34.81%, 34.93–54.21%, and 38.55–48.19% for *R. stolonifer*, *A. niger*, *E. coli* O157:H7, and *S. aureus*, respectively. The antimicrobial mechanism of AgNPs is associated with membrane disruption due to the release of positively charged silver ions that interact with the negatively charged cell membrane [21]. Furthermore, AgNPs can agglomerate surrounding the microbial cell membrane, generating reactive oxygen species (ROS) that target cellular enzymes responsible for cellular processes through the membrane [3]. Additionally, it was previously reported that AgNPs penetrate fungal spores and inhibit the sporulation of *R. stolonifer* as a result of DNA damage [76]. Zhang et al. [24] also observed that the addition of AgNPs to PLA packaging films successfully lowered the total number of microorganisms in strawberries, increasing fruit preservation by 10 days. The IC of the PLA/G-EOs and (PLA/G-EOs + AgNPs) active films was significantly increased by the addition of EOs (*p* ≤ 0.05). EOs are known for their antimicrobial effect against a wide spectrum of bacteria and fungi due to their hydrophobic characteristics that allow them to cross the cell wall, disrupting the balance and increasing permeability [77]. The observed antifungal effects align with earlier studies demonstrating that EOs rich in trans-cinnamaldehyde, thymol, and carvacrol disrupt fungal membranes and inhibit mycelial growth through multiple mechanisms, including increased membrane permeability [78]. Gonon et al. [54] observed that the addition of carvacrol and cinnamaldehyde (strong antifungal compounds present in oregano and cinnamon EOs) to PLA packaging trays resulted in strong antifungal activity, improving the shelf life of bakery products. Moreover, cinnamon EO can induce cell death by altering the distribution of lipids, destroying the cell membrane, and inhibiting ATP enzyme activity [79]. A recent study by Fang et al. [80] also found that the combined application of thyme and cinnamon EOs affected *E. coli* and *S. aureus* by interfering with bacterial DNA replication and repair.

### 3.2. Quality Parameters of Strawberries

#### 3.2.1. Visual Appearance and Decay Rate

**Figure 3:**
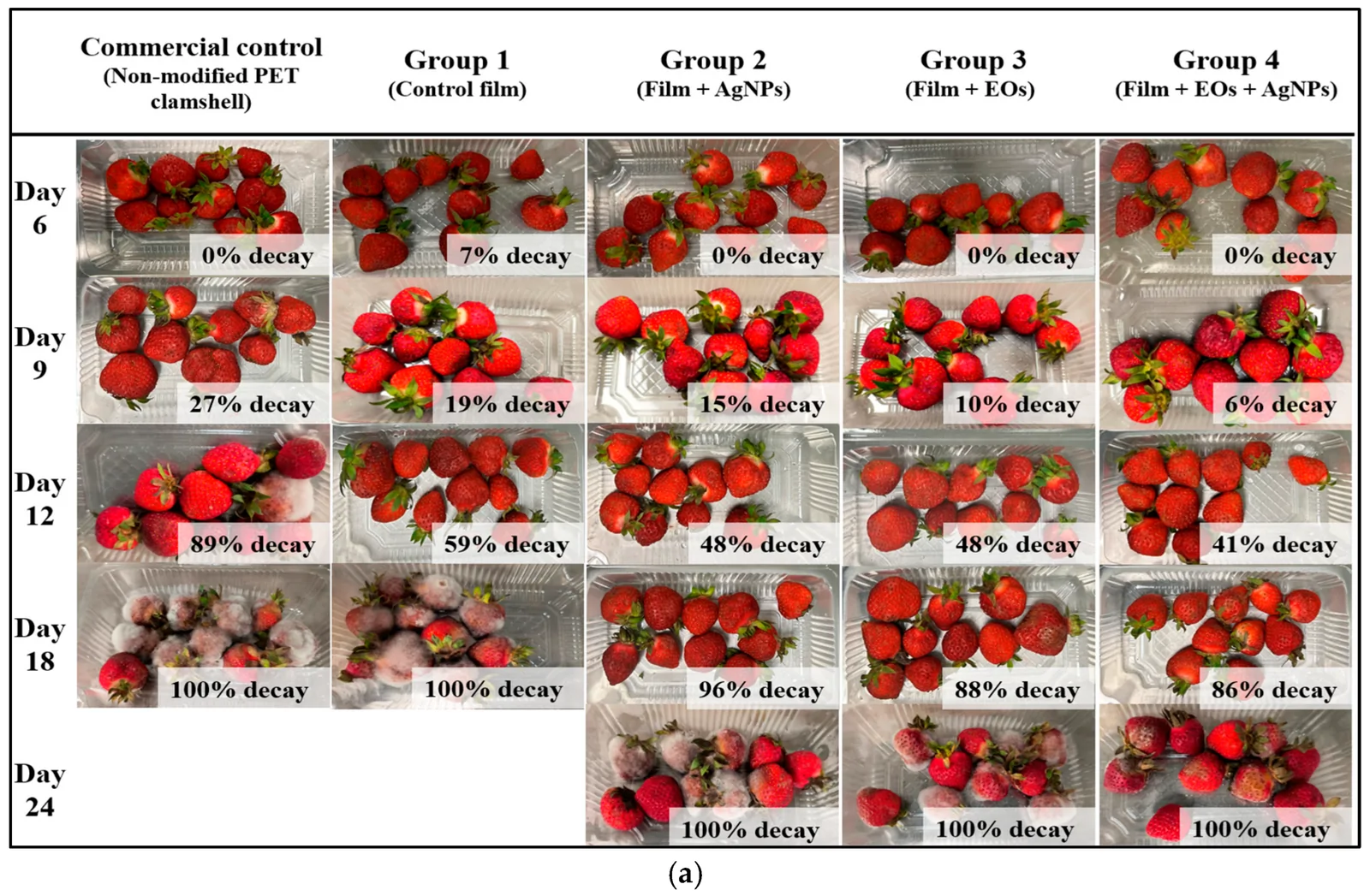

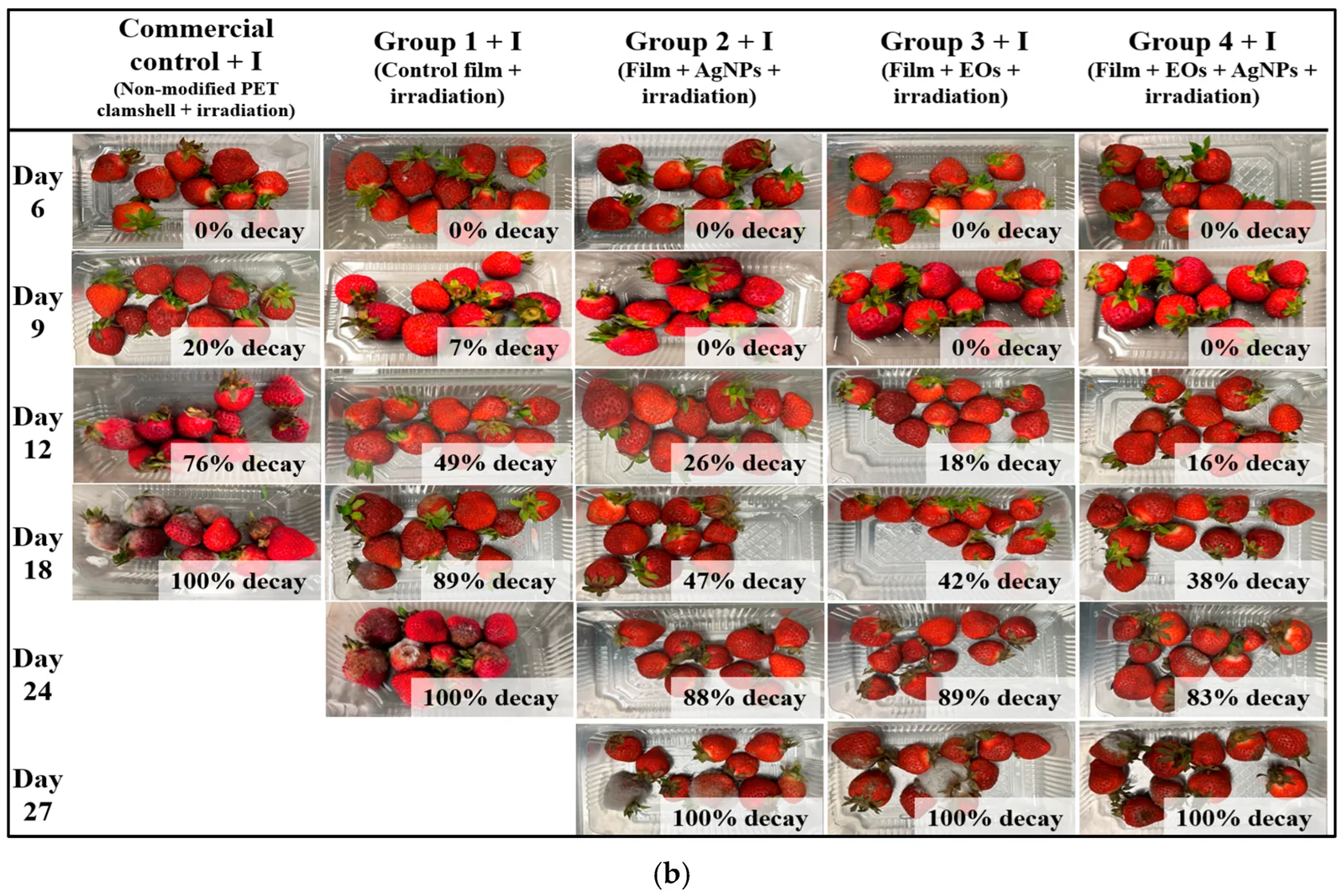
Decay rate and fruit appearance in packaging (**a**) and in packaging combined with L-E X-ray irradiation (**b**).

**Figure 4:**
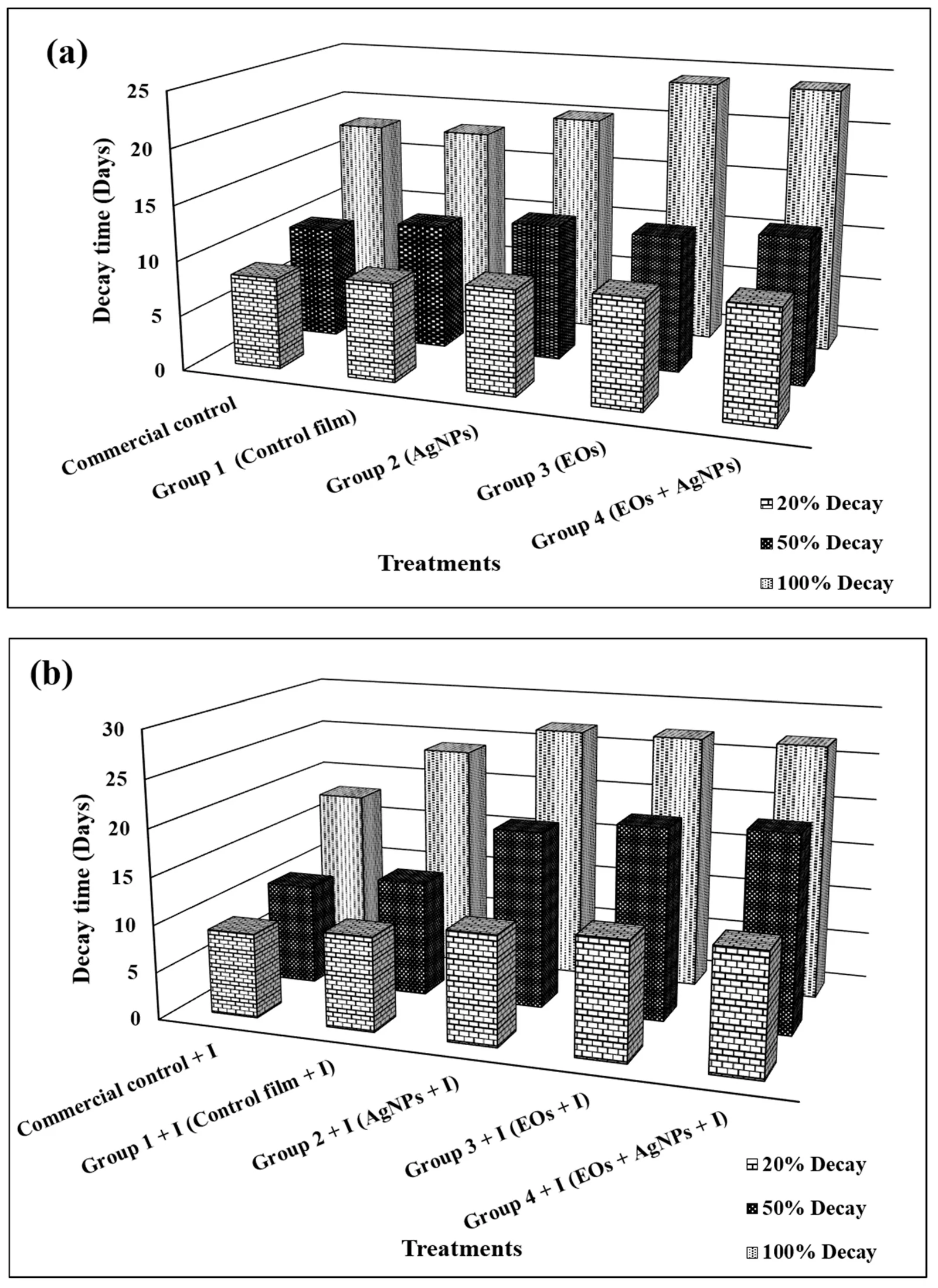
Strawberry decay time groups at 20, 50, and 100% deterioration in packaging (**a**) and in packaging combined with L-E X-ray irradiation (**b**).

The visual appearance and decay rate of strawberries treated with films alone and in combination with L-E X-ray irradiation are presented in Figure 3 and Figure 4 and Table 3. The results show that the application of active films significantly maintained the fresh appearance of strawberries and delayed visible decay during storage compared to the commercial control (non-modified PET clamshell) and Group 1 (PLA/G film) (Figure 3a). Fruit decay initially manifested through the appearance of localized softening and darkening on the surface of strawberries. Visible fungal growth became evident by day 12 in the commercial control. In contrast, visible signs of molds were delayed until day 18 for Group 1 (PLA/G film and postponed until day 24 in the samples packaged with active films, namely Group 2 (PLA/G-AgNPs), Group 3 (PLA/G-EOs), and Group 4 (PLA/G-EOs + AgNPs). Commercially available PET exhibits higher water and oxygen barrier properties [81] in comparison to the developed PLA-based films in the present study (OTR from 219–299, cm^3^/m^2^·day WVP from 1.70–1.93 g·mm/m^2^·day·kPa (Table 1). These findings highlight the potential advantage of higher WVP values in preventing internal moisture accumulation, thereby reducing the risk of water condensation that might promote fungal growth [14]. Additionally, an increase in the OTR facilitates O_2_ permeation into the package, which is critical to prevent anaerobic respiration and further spoilage [56]. The antimicrobial agents inside of the film matrix have an effect on the decay rate of strawberries. The film-embedded silver nanoparticles can suppress microbial growth on the packaging’s surface and in the headspace, creating a protective microenvironment that suppresses microbial proliferation without direct contact between antimicrobial agents and fruit surfaces, reducing the microbial load of the fruit and slowing spoilage [82]. According to the current food contact regulations for materials containing AgNPs, we believe that a ‘window of opportunity’ for safe and effective direct antifungal action via silver migration from the films to the fruit surface is unlikely to exist. The MIC of AgNPs against *Rhizopus stolonifer* and *Botrytis cinerea* (common fungi associated with strawberry) is 7.8–12.5 mg/L according to a recent study [83], whereas the specific migration limit for silver is 0.05 mg/kg food or mg/L food simulant, consistent with the EFSA’s safety assessment of AgNPs in food contact plastics. This comparison reveals a substantial gap between the silver concentration required for direct fungicidal activity against the molds and the legally permitted migration limit into food.

The antimicrobial activity of the volatile EOs helps reduce the microbial load and the decay of strawberries by restricting not only the pathogenic and spoilage bacteria but also fungi growth, as well as a slow release of active EO components throughout storage time. However, the level of EOs’ release from the film to the food depends on many factors, such as the film’s structure, storage conditions, and the food’s composition. Previous studies have shown that EOs can be stabilized in the film matrix when added in nanoemulsion form or encapsulated in the film matrix. This results in improving the antimicrobial properties of the film but also reduces the release of the antimicrobial agents from the film to the food [84,85].

The effect of L-E X-ray irradiation on strawberries had a significant impact on delaying fungal growth (Figure 3b). Visible fungal growth was observed by day 18 on the commercial control + I (PET clamshell + irradiation) and Group 1 + I (PLA/G film + irradiation), in contrast with the samples packaged with bioactive films (Group 2 + I, Group 3 + I, Group 4 + I), in which fungal growth was not observed before day 24. Inhibition of fungal growth after high-energy X-ray irradiation was previously reported by Yoon et al. [30], who observed that a dose of 1 kGy had significant effect on reducing the decay rate by 44.3% compared to the control sample (decay rate of 79.9%) after 9 days of storage at 15 °C. Compared to their non-irradiated counterparts, samples packaged with active films in combination with irradiation showed improved visual appearance and lower decay.

Overall, the developed active packaging systems effectively reduced the decay rate of strawberries. As shown in Figure 4a, the time required to reach 20% decay (primary criterion for shelf life) was approximately 8 days for the commercial control, increasing slightly to 9 days for Group 1 (PLA/G control film) and Group 2 (PLA/G-AgNPs), and 10 days for strawberries stored in Group 3 (PLA/G-EOs) and Group 4 (PLA/G-EOs + AgNPs). The application of L-E X-ray irradiation further extended this decay threshold (Figure 4b). Indeed, in irradiated samples, 20% decay was observed after 9 days for commercial control + I, 10 days for Group 1 + I (irradiated PLA/G control film), 11 days for Group 2 + I (irradiated PLA/G-AgNPs), 12 days for Group 3 + I (irradiated PLA/G-EOs), and 13 days for Group 4 + I (irradiated PLA/G-EOs + AgNPs). These results show a progressive improvement with simultaneous application of bioactive films and irradiation.

According to previous studies, the maximum decay threshold assessed from analysis of consumer willingness to purchase and deterioration data was 13% [86]. Other studies reported acceptable thresholds up to 10% for severe damage and up to 3% for mold incidence [87]. However, the 20% threshold was also considered acceptable according to another study [88]. From a market point of view, a higher decay % than 20% would not be acceptable for consumers. Yet, it was essential to complete the analyses (for the unaffected strawberries) even after exceeding the commercial threshold to have a better understanding of the biochemical and physiological changes of the strawberries as affected by the active films and L-E X-ray irradiation.

This trend is also observed for 50% decay thresholds and for the limit of 100% decay. Regarding the 50% decay threshold, the protective effect of the active films became more evident (Figure 4a). In the commercial control, 50% decay was reached by day 10, whereas it was delayed to 11 days in Group 1 (PLA/G control film), 12 days in Groups 2 and 3 (PLA/G- AgNPs and PLA/G-EOs), and 13 days in Group 4 (PLA/G-EOs + AgNPs). These results highlight the antifungal activity of EOs and AgNPs, particularly when both agents are combined. In combined treatment with X-ray irradiation (Figure 4b), the time to reach 50% decay further increased. For the commercial control + I (irradiated commercial clamshell) and Group 1 + I (irradiated PLA/G film), 50% decay was observed after 11 and 12 days, respectively, representing a 1-day extension in each treatment. In contrast, X-ray irradiation had a substantial effect when combined with AgNPs or EOs. Group 2 + I (irradiated PLA/G-AgNPs) reached 50% decay after 19 days, while Group 3 + I (irradiated PLA/G-EOs) further extended it to 20 days. The greatest delay was observed in Group 4 + I (irradiated PLA/G-EOs + AgNPs), where 50% decay was reached after 21 days, more than double the time compared to the non-irradiated commercial control. X-ray irradiation treatment exhibited a statistically significant effect (*p* ≤ 0.05), suggesting that irradiation had enhanced activity when applied together with EOs and/or AgNPs. Irradiation’s effect is related to its ability to destroy spoilage microorganism, especially on the surface of strawberry, hence preventing or minimizing the decay process [89].

Finally, complete decay (100%) was observed after 18 days for both irradiated and non-irradiated commercial controls and Group 1 (PLA/G control film) (Table 3). A slight increase was observed for Group 2 (PLA/G-AgNPs), where total decay was observed after 20 days. A more pronounced intermediate effect was observed in Group 3 (PLA/G-EOs), Group 4 (PLA/G-EOs + AgNPs), and Group 1 + I (PLA/G control film irradiated), where 100% decay was delayed until 24 days. When X-ray irradiation was applied in combination with antimicrobial films, the effect on shelf life was further amplified as result of the effect of antimicrobial EOs and AgNPs with irradiation [21]. Thus, Group 2 + I (irradiated PLA/G-AgNPs), Group 3 + I (irradiated PLA/G-EOs), and Group 4 + I (irradiated PLA/G-EOs + AgNPs) reached 100% decay after 27 days, marking the maximum delay observed among all treatments. These results agree with Shankar et al. [35], who observed that the combination between EOs and AgNPs in a chitosan packaging film in combination with gamma irradiation reduced decay to around 30% after 12 days of storage, demonstrating that the combined treatment was a successful technique to extend the shelf life of strawberries.

#### 3.2.2. Weight Loss

**Figure 5:**
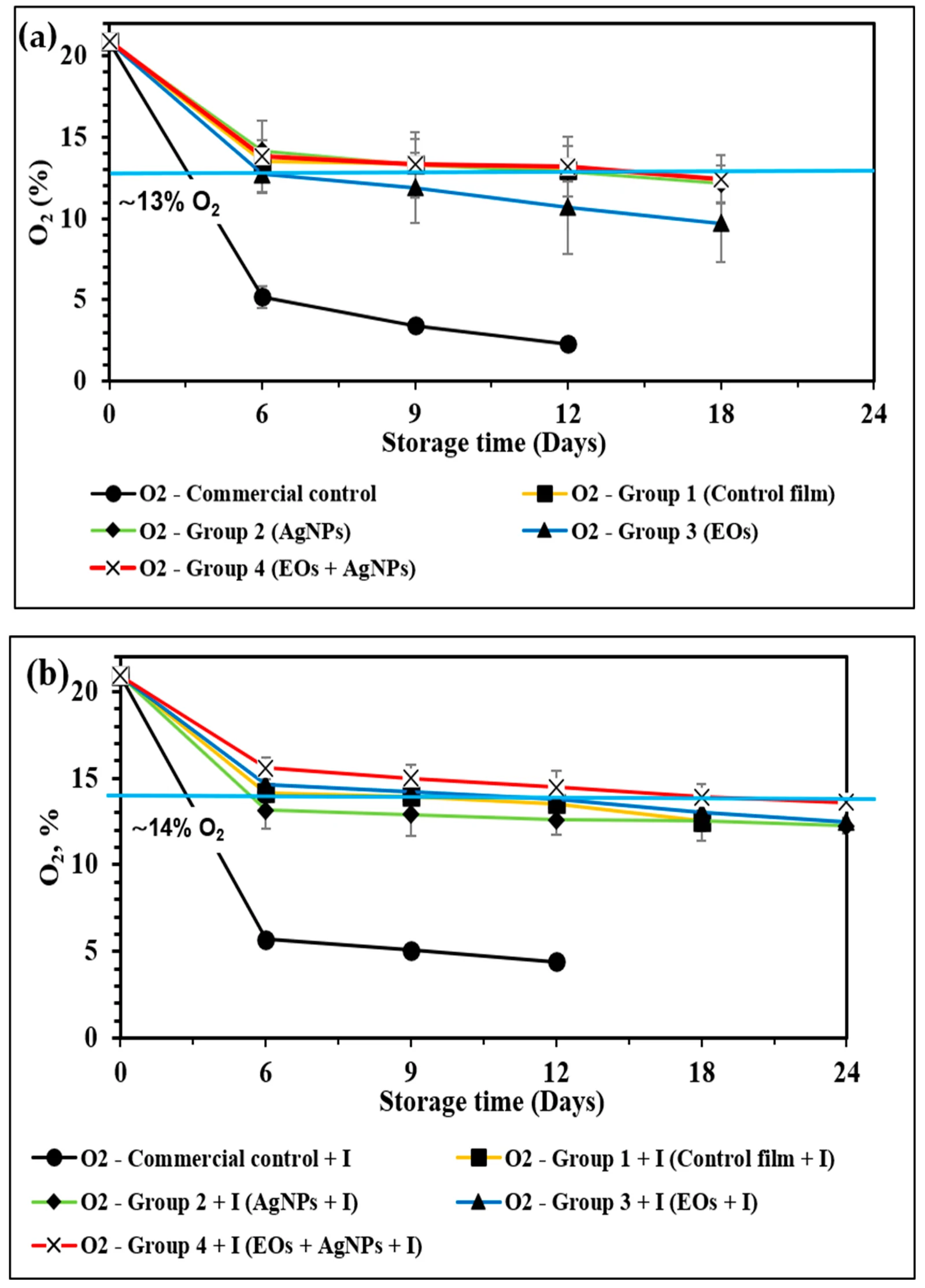

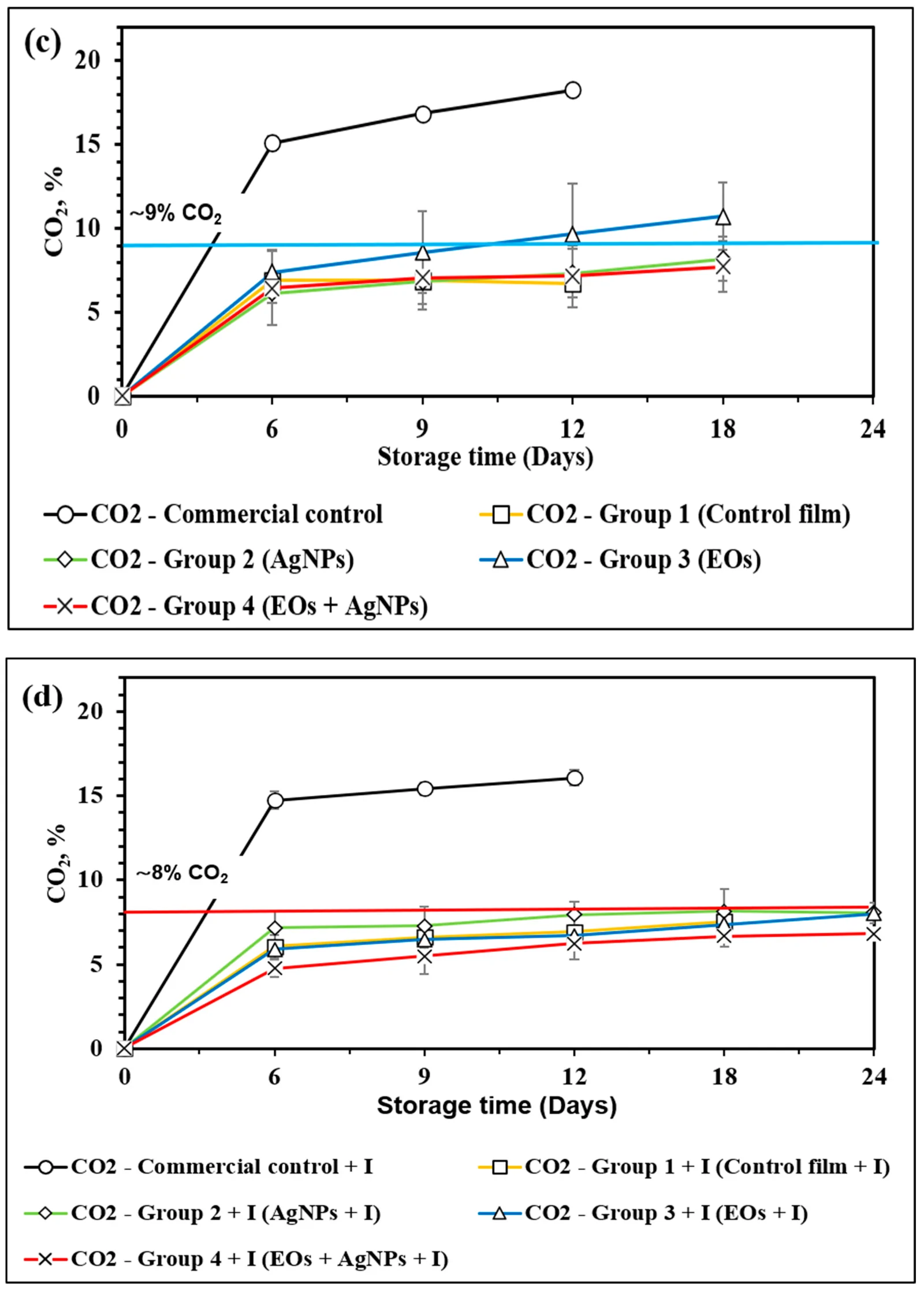
O_2_ composition inside of the strawberry package (**a**) and in the package combined with L-E X-ray irradiation (**b**). CO_2_ composition inside of the strawberry package (**c**) and in the package combined with L-E X-ray irradiation (**d**).

The effect of packaging treatments and L-E X-ray irradiation on the weight loss of strawberries is depicted in Table 3. Weight loss mainly causes deterioration of fruits in the form of wilting, shriveling, and softness and affects their salability. The weight loss measured during the total storage time was lower than 6% for all samples, which is the standard value to prevent loss of texture and shriveling in strawberries. Weight loss is the consequence of carbon loss due to respiration and water loss from the transpiration of the strawberries [90]. In general, X-ray combined treatment resulted in significantly lower weight loss (*p* ≤ 0.05) compared to non-irradiated counterparts over the same storage period, which could be due to the decreased respiration rate and reduced metabolic activity [89,91]. In addition, the gas and water permeability of the films played an important role in promoting adequate atmospheric conditions for strawberry preservation. Although the films exhibited higher water vapor and gas permeability compared to conventional PET packaging, their permeability characteristics were suitable for establishing an equilibrium-modified atmosphere for strawberry preservation (Figure 5). These conditions effectively minimized fruit dehydration and weight loss while also preventing excessive moisture accumulation within the package.

#### 3.2.3. Headspace Gas Composition

##### Oxygen Content

The headspace gas composition inside of packaging is the result of the relationship between the film’s permeability and the respiration rate of the strawberries during storage [92]. This interaction creates a modified atmosphere that eventually reaches a steady state described as equilibrium-modified atmosphere packaging (EMAP) by previous authors [57]. The improper control of gas compositions inside of strawberry packages may result in undesirable consequences, such as anaerobic respiration, accelerated decay, and short shelf life. The changes in the O_2_ content inside of the packaging during storage for non-irradiated and irradiated treatments are represented in Figure 5a,b, respectively. A sharp decrease in O_2_ level was observed during the first 6 days for the commercial control, from 21 to 5.3% O_2_, due to the low oxygen permeability of the PET. The O_2_ concentration further decreased to 2.27% O_2_ on day 12. Conversely, the O_2_ concentration inside of all of the PLA/G packaging groups was significantly higher (*p* ≤ 0.05) than in the commercial control due to higher O_2_ permeability, which allowed the permeation of O_2_ inside of the packaging. The O_2_ concentration decreased at a slower rate in these groups, with the same trend of a sharp decrease during the first 6 days of storage, followed by a steady state. As observed, O_2_ concentration within the packaging reached equilibrium by day 6, in which no statistically significant differences (*p* > 0.05) were detected until the end of storage. In particular, the O_2_ equilibrium plateau of Group 3 (PLA/G-EOs) was lower (~12.8–10.0%) than the other films (~13.8–12.5%). Comparable findings have been described by several authors, demonstrating that O_2_ concentrations ranging from 8 to 14% effectively preserve strawberries during storage [14,93].

For the irradiated samples (Figure 5b), a similar pattern of decrease was observed during the first 6 days of storage in the commercial control + I (irradiated PET clamshell) from 21 to 6% and further decreased to 4% O_2_ by day 12. The average O_2_ concentration for Group 1 + I (irradiated PLA/G film) and Group 3 + I (irradiated PLA/G-EOs) was slightly higher (around 14%) compared to Group 2 + I (irradiated PLA/G-AgNPs), which exhibited an O_2_ concentration of around 13% during the total time of storage. The highest O_2_ concentrations were observed in Group 4 + I (PLA/G-EOs + AgNPs + I), which ranged from 16% at day 6 to 14.2% after 24 days of storage, showing the slowest decrease in O_2_ concentration. In the PLA/G packaging systems, O_2_ concentrations ranging from 10 to 16% were maintained at a high plateau level during storage, which contributed positively to strawberry preservation when compared to the commercial control + I, in which O_2_ levels dropped rapidly below 4%. These results align with previous findings indicating that O_2_ content below 5% can cause anoxia in strawberries, resulting in fermentation of the fruit, causing off-odors, accelerating decay, and shortening shelf life [94].

##### Carbon Dioxide Content

Changes in the CO_2_ content of strawberry packaging for non-irradiated samples are depicted in Figure 5c and for irradiated samples in Figure 5d. Inversely to O_2_ as a respiration result, CO_2_ increased sharply during the first 6 days of storage. The CO_2_ content for commercial control increased from atmospheric conditions (0.04%) to 15% due to the high respiration rate of strawberries (between 20 and 40 mg·CO_2_·kg^−1^·h^−1^) [95] and the low CO_2_ permeability of PET compared with PLA [96]. The final CO_2_ concentration was 18.2% for the commercial control after 12 days of storage. The CO_2_ concentration inside of the PLA/G systems was significantly lower (*p* ≤ 0.05) than the commercial control due to the higher CO_2_ permeability of the PLA/G biopolymer film, which allowed the permeation of CO_2_ outside of the packaging. A rapid increase in CO_2_ concentration during the first 6 days was followed by a steady equilibrium state until the end of storage in Group 1 (PLA/G-control film), Group 2 (PLA/G-AgNPs), Group 3 (PLA/G-EOs), and Group 4 (PLA/G-EOs + AgNPs), where no significant differences were observed in the CO_2_ concentrations from day 6 to the end of storage (*p* > 0.05). The average CO_2_ concentrations measured with these films ranged between 7 and 9.5%. Irradiation slightly decreased CO_2_ levels. The commercial control + I (irradiated PET clamshell) exhibited an initial CO_2_ concentration of 15% on day 6, which slowly increased to 16% by day 12. On the other hand, the average CO_2_ levels for all PLA/G films equilibrated up to a lower plateau at 6.8–8.0% after 24 days of storage, with no significant difference between them (*p* > 0.05). High CO_2_ values (>30%) have a negative impact on strawberries, suppressing aerobic respiration, color, and texture changes. The recommended limit ranges between 10 and 20%, which can have a fungistatic effect against *B. cinerea* and *R. stolonifer* [4].

From these results, L-E X-ray irradiation was found to influence the headspace gas composition of strawberries’ packaging. Specifically, irradiated samples exhibited significantly higher O_2_ and lower CO_2_ concentrations compared to the non-irradiated samples (*p* ≤ 0.05) (Figure 5c,d). This reflects the impact of irradiation on respiration rate, as it slows the metabolism of strawberries, slowing the production of CO_2_ and hence slowing the consumption of O_2_ inside of the packaging [21,89]. Benyathiar et al. [97] reported that gamma irradiation up to 10 kGy did not significantly change the O_2_ and CO_2_ barrier properties of PLA films, ensuring the stability of the gas exchange in the irradiated packaging systems. The PLA/G nanocomposite developed in this study has successfully increased the O_2_ and CO_2_ permeability of the packaging system, which has been proven to be beneficial for strawberry preservation, preventing fruit anoxia. The developed PLA/G systems successfully maintained EMAP, which considers not only the natural respiration of the fruit but also the role packaging permeability plays in the atmospheric conditions inside of the packaging, allowing it to reach an optimum equilibrium. This technique, if appropriate, can decrease the cost of production, as the initial packaging of the fruit can be performed in atmospheric conditions (20.9% O_2_ and 0.03% CO_2_) that will be naturally modified by the respiration of strawberries and the movement of gases in and out of the packaging, therefore broadening its applicability [56]. In general, the in-package O_2_/CO_2_ atmosphere composition remained within the aerobic range throughout storage in all treatments, indicating that conditions of a hypoxic environment favorable for fermentative metabolism and subsequent associated off-odors/off-flavors were not formed. In addition, severe off-odors/off-flavors were not detected during handling or measurement studies of the treated strawberries throughout the in situ experiment by the research team at any sampling point.

#### 3.2.4. pH

The pH of strawberries can vary depending on the harvest time and fruit variety; it typically ranges between 3 and 3.9. The pH of strawberries during storage is shown in Table 3. The pH of the commercial control strawberry increased from 3.43 to 3.51 by day 6 and remained stable until the end of the storage. The use of the developed packaging delayed the increase in pH. For instance, Group 1 (PLA/G control film) maintained a pH of around 3.4 during 12 days of storage. A slight increase was observed in Group 2 (PLA/G- AgNPs), Group 3 (PLA/G-EOs), and Group 4 (PLA/G-EOs + AgNPs), with final values between 3.50 and 3.57 after 18 days of storage. The pH was increased due to the loss of water-soluble organic acids (vitamin C) during storage [98]. Additionally, it has been reported that higher CO_2_ levels in the packaging headspace can increase the pH of strawberries [57]. The treatment with low-energy X-ray did not have strong impact on the pH of strawberries compared to the non-irradiated samples. A slight increase was observed in all of the irradiated samples, regardless of treatment. The average pH was of 3.5 for Group 2 + I (PLA/G- AgNPs irradiated), Group 3 + I (PLA/G-EOs irradiated), and Group 4 + I (PLA/G-EOs + AgNPs irradiated) after 24 days of storage. These results demonstrate that the developed PLA-based packaging systems can maintain the acidity of strawberries, an important parameter related to the flavor of the fruits.

#### 3.2.5. Surface Color

The color of strawberries directly affects the fruit’s appeal and consumer acceptance. Changes in the surface color of strawberries were analyzed by measuring the lightness (L*), the redness (a*), and the yellowness (b*) of the fruits during storage, and the results are presented in Table 4. On day 0, the L*, a*, and b* values of strawberries were not significantly affected (*p* > 0.05) by irradiation treatment, with average values of L* = 32.2–34.9, a* = 26.6–27.2, and b* = 24.1–24.3. Results show that the L* values for all treatments remained stable during storage time, with no significant effects (*p* > 0.05) of the films’ treatments compared to the control. It has previously been reported that gamma and electron beam (e-beam) irradiation at a dose of 1 kGy did not affect the L* values of strawberries during storage [35,98].

In addition, an increase in the redness of irradiated strawberries was observed between day 6 and day 9, as indicated by the rise in a* values from 26.64 for the non-irradiated samples to 27.1–30.30 depending on the treatments; the highest values were obtained in irradiated Groups 1 and 2, with values of 30.30 and 29.84 at days 6 and 9, respectively, in line with the results of Yoon et al. [34]. The increase in a* values in the surface of the strawberries may be attributed to irradiation-induced activation of phenylalanine ammonia-lyase (PAL), which is involved in anthocyanin synthesis [99]. Afterwards, the redness of all of the samples decreased during storage, which may be due to enzymatic degradation of anthocyanin by hydrolytic enzymes (such as polyphenol oxidase (PPO)) of fruits, which affects the color of fruit [24,35]. PPO catalyzes the reaction of polyphenol compounds with oxygen, resulting in the formation of quinones that react with anthocyanins, producing brown compounds and causing discoloration of anthocyanins [100]. The yellowness values of strawberries of all of the samples had a slight decrease during storage, which exhibited an initial b* average value around 24 (day 0) and then experienced a decrease with storage progression, reaching average values between 20.30 and 23.17 on days 12–18 for the non-irradiated samples and between 18.74 and 21.39 on days 18–24 for the irradiated samples. Irradiation did not affect the yellowness of the samples significantly (*p* > 0.05), which exhibited comparable values to the non-irradiated strawberries. These results align with Barkaoui et al. [93], who observed that strawberries treated through e-beam irradiation at doses up to 2 kGy displayed b* values similar to the non-irradiated fruits.

#### 3.2.6. Firmness

The maximum penetration force (N) corresponding to the epidermis firmness of strawberries is shown in Table 5. Strawberry firmness is greatly influenced by environmental conditions such as temperature, carbon dioxide, and light exposure. Among other parameters, epidermis firmness is one of the main parameters used to predict the shelf life of the fruit. This parameter shows a strong negative correlation with weight loss in all groups (r between −0.76 and −0.99), which agrees with results found by Pasha et al. [101], who suggest that firmness loss is related to water loss of the fruit. The firmness of all samples decreased rapidly during the first 6 days of storage, specifically for the commercial control (non-modified PET clamshell), in which the firmness decreased from 0.78 to 0.50 N. At day 6 of storage, no difference in firmness was observed between Group 1 (PLA/G control film), Group 2 (PLA/G-AgNPs), and Group 3 (PLA/G-EOs), with an average firmness of 0.51 N, while Group 4 (PLA/G-EOs + AgNPs) maintained a firmness of 0.62 N. Strawberry softening is part of the senescence process, and it can be retarded by lowering the respiration rates of the strawberries [102]. Fungal infection, mainly caused by B. cinerea and R. stolonifer, can also significantly contribute to the fruit softening due to the activity of enzymes such as polygalacturonases, xylanase, and cellulase [103].

Initial firmness of irradiated strawberry at day 0 was not significantly affected after low-energy X-ray irradiation, recording an average value of 0.78 N. Low-energy X-ray reduced the loss of the firmness of strawberries during storage. After 6 days of storage, Group 1 (PLA/G control film irradiated) exhibited higher firmness (0.60 N) than the commercial control + I (irradiated) (0.54 N). The samples treated with antimicrobial compounds and low-energy X-ray showed an average firmness of 0.67 N, the highest among all treated samples. The groups with antimicrobial agents in combination with irradiation had higher firmness compared to the non-irradiated samples. Group 2 + I (PLA/G-AgNPs irradiated), Group 3 + I (PLA/G-EOs irradiated), and Group 4 + I (PLA/G-EOs + AgNPs irradiated) maintained the highest firmness, with mean values around 0.67 N by day 6, 0.62 N by day 9, 0.58 N by day 12, and 0.52 N by day 18. The combination of an antimicrobial agent (AgNPs and/or EOs) and low-dose X-ray irradiation of these groups helped preserve the firmness of the fruits through storage. These results indicate that the combined treatment helped maintain the original texture of strawberries during storage and additionally exhibited a remarkably lower decay rate.

#### 3.2.7. Total Soluble Solids (TSS)

TSS content is strawberry quality indicator of maturity and quality, and changes during storage. Table 5 shows the effects of the developed PLA-based packaging films, low-energy X-ray irradiation, and storage time on the TSS of strawberries. No statistical difference (*p* > 0.05) was observed between the groups during the first 6 days of storage with an average value between 7.93–8.07 °Brix for the Commercial control, Group 1 (PLA/G-EOs + AgNPs control film), Group 2 (PLA/G- AgNPs), Group 3 (PLA/G-EOs), and Group 4 (PLA/G-EOs + AgNPs). All the treatments experienced an increase in TSS value, followed by a decrease at day 12–18 of storage. The increase in TSS is due to the inherent metabolic processes of strawberries convert carbohydrates of cell wall mainly, into sugars and soluble compounds [104]. By day 12, a decrease in the TSS was observed in the Commercial control (7.07 °Brix), while the samples treated with the developed packaging exhibited TSS of between 7.93–8.33 °Brix. The subsequent decrease in TSS with storage because the sugars are used as a source of energy during metabolic processes. Other authors have reported an initial increase of the TSS during the first 6 to 12 days as a result of water loss of the fruit or the conversion of starch and other organic acid such as ascorbic acid into sugar [65]. After this period, a slight decrease in the TSS was observed in all the samples.

No significant difference (*p* > 0.05) was observed in the TSS in irradiated strawberries during the first 9 days of storage. These results agree with Yoon, et al. [30], who didn’t observe any change in the TSS of strawberries after irradiation at a dose of 1 kGy using high-energy X-ray. The combined treatment of Group 2 + I (PLA/G- AgNPs irradiated), Group 3 + I (PLA/G-EOs irradiated), and Group 4 + I (PLA/G-EOs + AgNPs irradiated) exhibited a significant higher TSS content compared to the rest of the treatments, with an average of 8.00 °Brix after 18 days of storage. These results demonstrate that the combination of antimicrobial agents (AgNPs and/or EOs) with low-energy X-ray irradiation can preserve the sugar content of strawberries and therefore, its quality for a prolonged period of time as a result of the reduction in the respiration rate of strawberries slowing their senescence [101].

#### 3.2.8. Biochemical Analyses

##### Total Phenolic Content (TPC)

Total phenolic compounds are important nutritional components that contribute not only to the flavor of strawberries but also to their color and antioxidant properties, which make strawberries a valuable fruit. The change in the TPC in strawberries during storage is shown in Table 5. The initial TPC was 201.6 mg GAE/100 g on day 0 for the non-irradiated group. A statistically significant increase (*p* ≤ 0.05) in TPC was observed after low-energy X-ray irradiation. Specifically, the TPC of non-irradiated samples exhibited higher content of 208.1 mg GAE/100 g (*p* ≤ 0.05). A slight increase in TPC occurred in all groups between 6 and 9 days of storage, followed by a decrease in TPC. The highest increase was observed in Group 1 (PLA/G- control film) to 224.5 mg GAE/100 g after 6 days of storage and in irradiated Group 1 (from 208.1 to 244.2 mg GAE/100 after 6 days of storage (*p* ≤ 0.05). The increase in phenolic compounds is associated with multiple factors; some authors describe it as a stress response to environmental factors such as irradiation exposure, temperature, and atmospheric conditions [105]. Alwazeer and Ozkan [15] reported that atmospheric composition, especially higher CO_2_ content, has a protective effect on TPC in strawberry packaging. A gradual decrease in TPC was observed in all groups until the end of storage. The samples treated with PLA/G-EOs maintained statistically higher TPC than the other treatments after 18 days of storage, with an average value of 179.6 mg GAE/100 g. The highest TPC at day 6 was found in Group 1 + I (PLA/G-film irradiated; 244.2 mg GAE/100 g), followed by Group 4 + I (PLA/G-EOs + AgNPs irradiated; 227.3 mg GAE/100 g), indicating a strong effect of low-energy X-ray irradiation on phenolic compound accumulation. Previous studies have observed an increase in TPC after different irradiation treatments, which is closely related to the increase in the activity of the phenylalanine ammonia-lyase (PAL), an enzyme that is related to the biosynthesis of phenolic compounds [99,106].

Over time, a gradual decline in TPC was observed across all treatments, reflecting phenolic degradation during storage. However, irradiated samples generally maintained slightly higher TPC values compared to the non-irradiated strawberries. For instance, at day 12, Group 3 + I (PLA/G-EOs irradiated) retained 190.4 mg GAE/100 g versus 163.3 mg GAE/100 g in Group 3 (PLA/G-EOs), and, by day 24, Group 2 + I (PLA/G-AgNPs irradiated) and Group 4 + I (PLA/G-EOs + AgNPs irradiated) still exhibited elevated TPC levels (192 and 167 mg GAE/100 g, respectively). These results demonstrate the positive effect of low-energy X-ray irradiation on strawberries to maintain TPC stability over time.

##### Anthocyanin Content

Anthocyanins are flavonoids that confer the red color to strawberries. Anthocyanin content was initially increased by irradiation, as observed in Table 5. There was a trend of increasing anthocyanin content during storage despite treatment, especially between 6 and 12 days of storage. Group 1 (PLA/G control film) exhibited the highest anthocyanin content of 154.7 mg CGE/L after 12 days of storage, as the biosynthesis pathway for anthocyanins is still active after harvesting [107]. After reaching peak values, a gradual decline was observed in all treatments, reflecting anthocyanin degradation; however, the final values for Group 2 (PLA/G-AgNPs), Group 3 (PLA/G-EOs), and Group 4 (PLA/G-EOs + AgNPs) ranged between 110 and 135 mg CGE/L, statistically higher (*p* ≤ 0.05) than the initial value (103.3 mg CGE/L). The treatment with low-energy X-ray irradiation significantly increased anthocyanin content to 109.1 mg CGE/L compared to 103.3 mg CGE/L in non-irradiated samples (*p* ≤ 0.05). This increase is related to the upregulation of PAL and glucosyltransferase (GL), key enzymes in the anthocyanin biosynthesis pathway induced by irradiation [91,108]. By day 9, the commercial control + I (irradiated) exhibited the highest anthocyanin concentration (173.5 mg CGE/L), followed by Group 1 + I (PLA/G film irradiated) with 169.1 mg CGE/L, highlighting a consistent enhancement of anthocyanin accumulation in irradiated systems. By day 24, irradiated groups such as Group 2 + I (PLA/G- AgNPs irradiated), Group 3 + I (PLA/G-EOs irradiated), and Group 4 + I (PLA/G-EOs + AgNPs irradiated) retained high anthocyanin content of 155.2, 132.1, and 113.8 mg CGE/L, respectively, which was higher than their initial levels. These findings suggest that low-energy X-ray irradiation not only stimulated anthocyanin biosynthesis through the induction of PAL and GL enzyme activity but also contributed to pigment stability over time. This preservation aligns with the sustained a* values (Table 4) previously observed in irradiated groups, indicating that low-energy X-ray is able to maintain the red coloration of strawberries during extended storage.

##### Vitamin C Content

The changes in total ascorbic acid (TAA), ascorbic acid (AA), and dehydroascorbic acid (DHA) are presented in Table 6. A gradual decrease in the TAA, AA, and DHA of all samples during storage time was observed in all treatments. Vitamin C degradation occurs through enzymatic activity and non-enzymatic factors, with oxygen being one of the most important oxidation factors [109]. TAA, AA, and DHA during storage of all samples packaged with films were statistically higher than the commercial control samples, despite antimicrobial treatment (*p* ≤ 0.05). By day 12 of storage, the TAA of Group 1 (PLA/G control film), Group 2 (PLA/G-AgNPs), Group 3 (PLA/G-EOs), and Group 4 (PLA/G-EOs + AgNPs) ranged from 59 to 62 mg/100 g compared to the commercial control at 44 mg/100 g. The content of AA of the same groups was between 41 and 44 mg/100 g compared to the commercial control at only 32 mg/100 g by day 12. Group 3 (PLA/G-EOs) exhibited almost double the DHA (oxidized ascorbic acid) compared to the commercial control with 20 mg/100 g compared to 12 mg/100 g after 12 days of storage. According to Xueyan et al. [93], modified atmosphere conditions have been reported to result in better AA retention compared to air-stored strawberries with low O_2_. In addition, MAP conditions using 10% O_2_, 15% CO_2_, and 75% N_2_ were found to slow down the respiration rate and metabolic activity of strawberries during storage [10]. Previous studies have found that PLA packaging material with NPs such as AgNPs or TiO_2_ can preserve the freshness of strawberries and reduce ascorbic acid loss during storage due to an increase in PLA permeability, promoting a modified atmosphere inside of the package that slows the respiration rate and delays the oxidation of the ascorbic acid [110].

A significant reduction in the initial TAA content was observed following low-energy X-ray irradiation, decreasing from 84.3 mg/100 g for the non-irradiated samples to 79.8 mg/100 g in the irradiated strawberries (*p* ≤ 0.05). The same effect was observed in AA, which decreased from 50.9 mg/100 g to 44.7 mg/100 g after irradiation (*p* ≤ 0.05). However, the DHA increased by 3 mg/100 g. Because greater loss of TAA and AA is recorded in the commercial control treatment, it can be inferred that the main loss of AA may be greatly affected by storage time rather than irradiation, in line with the findings of Hussain et al. [106]. A gradual decrease in TAA, AA, and DHA was also observed in the irradiated samples without any remarkable difference compared to the non-irradiated samples. For instance, by day 12 of storage, the TAA of Group 1 (PLA/G control film irradiated), Group 2 + I (PLA/G-AgNPs irradiated), Group 3 + I (PLA/G-EOs irradiated), and Group 4 + I (PLA/G-EOs + AgNPs irradiated) ranged from 59 to 64 mg/100 g compared to 58–52 mg/100 g for the same treatments without irradiation. These results suggest that the effect of the films had a greater influence on vitamin C content than irradiation. No significant decrease was observed in the initial DHA content of the irradiated samples (*p* > 0.05), which may be attributed to the effect of irradiation, as it has been reported to induce the oxidation of AA into DHA. Despite this reversible conversion, DHA is still active biologically and does not diminish the nutritional value of strawberries [111]. Although low-energy X-ray irradiation initially reduced TAA and AA content, the effect on nutrient losses caused by low doses of irradiation (<1 kGy) has been reported to be insignificant [112].

## 4. Conclusions

This study demonstrated the effectiveness of the developed PLA/G-based bioactive films incorporating EOs and AgNPs with low-energy X-ray irradiation in maintaining strawberry quality and extending shelf life. The addition of AgNPs enhanced the thermal stability of the films without affecting mechanical or barrier properties, such as WVP and OTR. The PLA/G-based films with EOs and/or AgNPs showed antifungal activity against R. stolonifer, a key factor that in combination with low-energy X-ray significantly delayed fungal growth and maintained fruit quality during storage. The PLA/G-based packaging maintained optimal internal gas conditions (O_2_ 13–14% and CO_2_ < 10%), which effectively lowered the respiration rate and delayed senescence. These conditions improved the retention of firmness, color, TSS, and pH. Irradiated samples exhibited enhanced TPC and anthocyanin content, possibly due to irradiation-induced PAL enzyme activation. The total vitamin C content was maintained over time, possibly due to reduced metabolism. These findings highlight the effect of using PLA-based films containing EOs and AgNPs with low-energy X-ray in extending the postharvest shelf life of strawberries while maintaining their physicochemical and nutritional quality. Despite the promising results obtained in this study, some limitations should be acknowledged. The effect of irradiation alone was not addressed, which would allow us to evaluate the synergistic effect of the irradiation and packaging films using two-way ANOVA. In addition, migration studies of AgNPs and EOs were not investigated. Future work should address these gaps and evaluate the long-term performance of the developed packaging system under industrial storage conditions, which will provide a more comprehensive assessment of the safety and applicability of the developed films for food contact applications. Moreover, while the developed active films showed strong performance in enhancing the shelf life and quality of strawberries, a formal quantitative sensory evaluation by panelists is required for stored strawberries before further scale-up studies, in addition to determinations of ethylene concentration and film characterization, such as surface morphology. Overall, the developed PLA-based bioactive composite films offer a potential sustainable alternative to conventional packaging systems.

## Figures and Tables

**Table 1:** Physicochemical, mechanical, and barrier properties of the films.

Films	WS (%)	Thickness (µm)	TS (MPa)	YM (MPa)	Eb (%)	WVP (g·mm/m^2^ · day·kPa)	OTR (cm^3^/m^2^·day)	WCA (°)
Control film (PLA/G)	1.18 ± 0.08 ^a^	149.40 ± 23.81 ^a^	16.70 ± 2.33 ^c^	503.40 ± 65.70 ^d^	215.40 ± 37.94 ^a^	1.74 ± 0.11 ^a^	219.77 ± 22.68 ^a^	69.66 ± 1.63 ^b^
PLA/G-AgNPs	1.15 ± 0.04 ^a^	122.20 ± 8.79 ^a^	10.01 ± 1.33 ^b^	292.80 ± 29.20 ^c^	218.20 ± 20.58 ^a^	1.90 ± 0.08 ^ab^	290.03 ± 23.45 ^b^	66.31 ± 1.59 ^c^
PLA/G-EOs	1.57 ± 0.03 ^b^	137.00 ± 12.37 ^a^	7.78 ± 0.98 ^a^	116.02 ± 22.69 ^b^	385.80 ± 39.05 ^b^	1.93 ± 0.09 ^b^	274.89 ± 23.75 ^b^	82.45 ± 2.08 ^a^
PLA/G-EOs + AgNPs	1.59 ± 0.06 ^b^	142.60 ± 18.15 ^a^	6.65 ± 0.87 ^a^	32.56 ± 19.91 ^a^	389.20 ± 28.12 ^b^	1.90 ± 0.01 ^ab^	298.67 ± 8.30 ^b^	81.35 ± 2.30 ^a^

Means ± standard deviation followed by the same letter within the same column are not significantly different according to Duncan’s multiple range test (*p* > 0.05). WS: water solubility; TS: tensile strength; YM: Young’s modulus; Eb: elongation at break; WVP: water vapor permeability; OTR: oxygen transmission rate; WCA: water contact angle.

**Table 2:** Inhibitory capacity (IC, %) of the PLA/G films.

Films	IC (%)			
	*R. stolonifer*	*A. niger*	*E. coli* O157:H7	*S. aureus*
Control film (PLA/G)	0.00 ± 0.00 ^a^	0.00 ± 0.00 ^a^	0.00 ± 0.00 ^a^	0.00 ± 0.00 ^a^
PLA/G-AgNPs	18.79 ± 4.15 ^b^	23.15 ± 2 ^b^	34.93 ± 2.50 ^b^	38.55 ± 1.15 ^b^
PLA/G-EOs	27.95 ± 1.64 ^c^	29.77 ± 1.27 ^c^	45.18 ± 4.23 ^c^	40.35 ± 1.50 ^b^
PLA/G-EOs + AgNPs	29.80 ± 2.83 ^c^	34.81 ± 2.29 ^d^	54.21 ± 1.75 ^d^	48.19 ± 3.17 ^c^

Means ± standard deviation followed by the same letter within the same column are not significantly different based on Duncan’s multiple range test (*p* > 0.05).

**Table 3:** Effect of packaging and irradiation treatments on the decay, weight loss, and pH of strawberries during storage.

Treatment	Day	Decay (%)	Weight Loss (%)	pH
Non-irradiated	0	0.00 ± 0.00 ^a^	0.00 ± 0.00 ^a^	3.43 ± 0.01 ^abc^
Commercial control	6	0.00 ± 0.00 ^a^	0.57 ± 0.06 ^klm^	3.51 ± 0.01 ^e–m^
	9	27.17 ± 6.91 ^f^	0.77 ± 0.04 ^n^	3.53 ± 0.04 ^h–m^
	12	89.39 ± 8.85 ^kl^	1.00 ± 0.05 ^p^	3.54 ± 0.01 ^h–m^
	18	100.00 ± 0.00 ^m^	-	-
Group 1 PLA/G	6	7.33 ± 8.43 ^abc^	0.35 ± 0.03 ^def^	3.43 ± 0.01 ^a–d^
	9	18.83 ± 11.30 ^de^	0.55 ± 0.02 ^jkl^	3.43 ± 0.08 ^a–d^
	12	58.50 ± 9.31 ^i^	0.74 ± 0.02 ^n^	3.46 ± 0.04 ^a–g^
	18	100.00 ± 0.00 ^m^	-	-
Group 2 PLA/G-AgNPs	6	0.00 ± 0.00 ^a^	0.23 ± 0.05 ^bc^	3.42 ± 0.01 ^ab^
	9	15.17 ± 7.57 ^cd^	0.39 ± 0.08 ^efg^	3.43 ± 0.01 ^a–d^
	12	48.33 ± 11.50 ^h^	0.54 ± 0.10 ^i–l^	3.44 ± 0.05 ^a–e^
	18	95.50 ± 7.31 ^lm^	0.97 ± 0.17 ^op^	3.50 ± 0.02 ^c–l^
	24	100.00 ± 0.00 ^m^	-	-
Group 3 PLA/G-EOs	6	0.00 ± 0.00 ^a^	0.24 ± 0.09 ^bc^	3.44 ± 0.04 ^a–f^
	9	9.50 ± 8.34 ^bc^	0.37 ± 0.08 ^d–g^	3.45 ± 0.01 ^a–g^
	12	48.33 ± 11.50 ^h^	0.51 ± 0.09 ^h–k^	3.50 ± 0.06 ^c–l^
	18	87.67 ± 7.28 ^kl^	0.90 ± 0.07 ^o^	3.57 ± 0.01 ^lm^
	24	100.00 ± 0.00 ^m^	-	-
Group 4 PLA/G-EOs + AgNPs	6	0.00 ± 0.00 ^a^	0.25 ± 0.05 ^bc^	3.47 ± 0.01 ^b–h^
	9	6.17 ± 7.22 ^ab^	0.39 ± 0.06 ^efg^	3.51 ± 0.02 ^e–m^
	12	41.00 ± 7.64 ^gh^	0.58 ± 0.05 ^klm^	3.52 ± 0.05 ^f–m^
	18	86.00 ± 7.77 ^k^	0.96 ± 0.06 ^op^	3.54 ± 0.06 ^h–m^
	24	100.00 ± 0.00 ^m^	-	-
Irradiated	0	0.00 ± 0.00 ^a^	0.00 ± 0.00 ^a^	3.39 ± 0.07 ^a^
Commercial control + irradiation (I)	6	0.00 ± 0.00 ^a^	0.28 ± 0.03 ^cd^	3.50 ± 0.02 ^d–l^
	9	19.50 ± 7.56 ^def^	0.45 ± 0.04 ^g–j^	3.56 ± 0.09 ^j–m^
	12	76.17 ± 9.58 ^j^	0.63 ± 0.04 ^lm^	3.56 ± 0.03 ^j–m^
	18	100.00 ± 0.00 ^m^	-	-
Group 1 PLA/G I	6	0.00 ± 0.00 ^a^	0.35 ± 0.04 ^def^	3.46 ± 0.03 ^a–g^
	9	7.33 ± 8.43 ^abc^	0.54 ± 0.06 ^i–l^	3.52 ± 0.05 ^g–m^
	12	48.83 ± 8.73 ^h^	0.77 ± 0.06 ^n^	3.53 ± 0.01 ^h–m^
	18	89.33 ± 8.89 ^kl^	1.21 ± 0.10 ^r^	3.56 ± 0.01 ^klm^
	24	100.00 ± 0.00 ^m^	-	-
Group 2 PLA/G-AgNPs + I	6	0.00 ± 0.00 ^a^	0.19 ± 0.01 ^b^	3.47 ± 0.01 ^b–h^
	9	0.00 ± 0.00 ^a^	0.32 ± 0.03 ^c–f^	3.48 ± 0.02 ^b–i^
	12	25.50 ± 7.29 ^ef^	0.45 ± 0.02 ^ghi^	3.48 ± 0.01 ^b–i^
	18	47.17 ± 12.97 ^h^	0.76 ± 0.01 ^n^	3.55 ± 0.05 ^i–m^
	24	87.67 ± 7.28 ^kl^	1.27 ± 0.06 ^r^	3.58 ± 0.03 ^m^
	27	100.00 ± 0.00 ^m^	-	-
Group 3 PLA/G-EOs + I	6	0.00 ± 0.00 ^a^	0.16 ± 0.03 ^b^	3.49 ± 0.01 ^b–h^
	9	0.00 ± 0.00 ^a^	0.30 ± 0.04 ^cde^	3.51 ± 0.01 ^e–m^
	12	17.83 ± 8.47 ^de^	0.41 ± 0.02 ^fgh^	3.51 ± 0.01 ^e–m^
	18	41.67 ± 9.83 ^gh^	0.74 ± 0.05 ^n^	3.52 ± 0.01 ^f–m^
	24	89.33 ± 8.89 ^kl^	1.09 ± 0.01 ^q^	3.52 ± 0.01 ^f–m^
	27	100.00 ± 0.00 ^m^	-	-
Group 4 PLA/G-EOs + AgNPs + I	6	0.00 ± 0.00 ^a^	0.31 ± 0.06 ^cde^	3.48 ± 0.04 ^b–j^
	9	0.00 ± 0.00 ^a^	0.45 ± 0.04 ^ghi^	3.49 ± 0.01 ^b–l^
	12	15.67 ± 10.05 ^cd^	0.65 ± 0.03 ^m^	3.51 ± 0.01 ^e–m^
	18	37.67 ± 7.28 ^g^	0.93 ± 0.03 ^op^	3.51 ± 0.04 ^e–m^
	24	82.67 ± 9.75 ^jk^	1.24 ± 0.04 ^r^	3.54 ± 0.05 ^h–m^
	27	100.00 ± 0.00 ^m^	-	-

In each column, means ± standard deviation followed by the same letter are not significantly different based on Duncan’s multiple range test (*p* > 0.05).

**Table 4:** Effect of packaging and irradiation treatment on the color parameters L* (lightness), a* (redness), and b* (yellowness) of strawberries during storage.

Treatment	Day	L*	a*	b*
Non-irradiated	0	34.89 ± 3.69 ^e–h^	26.64 ± 2.02 ^a–g^	24.28 ± 4.18 ^b–i^
Commercial control	6	33.17 ± 3.28 ^b–h^	29.04 ± 2.95 ^f–h^	24.79 ± 5.76 ^d–i^
	9	35.77 ± 2.91 ^f–h^	27.82 ± 2.44 ^c–h^	26.08 ± 4.00 ^f–i^
	12	31.70 ± 2.85 ^a–e^	27.06 ± 2.00 ^a–h^	20.30 ± 3.08 ^a–e^
Group 1 PLA/G	6	32.86 ± 3.64 ^a–h^	26.12 ± 3.22 ^a–g^	22.09 ± 4.48 ^a–g^
	9	33.61 ± 2.28 ^b–h^	25.21 ± 2.85 ^a–d^	22.06 ± 4.63 ^a–g^
	12	34.18 ± 3.20 ^b–h^	26.34 ± 2.72 ^a–g^	22.71 ± 5.20 ^a–h^
Group 2 PLA/G-AgNPs	6	32.91 ± 2.98 ^b–h^	27.32 ± 2.88 ^b–g^	23.08 ± 4.67 ^a–i^
	9	34.20 ± 3.90 ^b–h^	25.84 ± 3.75 ^a–g^	24.09 ± 5.84 ^b–i^
	12	32.84 ± 3.57 ^a–h^	24.86 ± 2.97 ^a–c^	20.61 ± 4.14 ^a–f^
	18	32.04 ± 2.31 ^a–f^	26.09 ± 1.78 ^a–g^	21.84 ± 3.54 ^a–g^
Group 3 PLA/G-EOs	6	36.11 ± 3.85 ^g–h^	29.17 ± 3.22 ^g–h^	27.12 ± 6.35 ^g–i^
	9	33.48 ± 2.76 ^b–h^	26.99 ± 1.98 ^a–g^	22.61 ± 4.89 ^a–h^
	12	33.30 ± 2.62 ^b–h^	25.03 ± 2.42 ^a–c^	20.12 ± 3.83 ^a–e^
	18	33.01 ± 4.36 ^b–h^	27.77 ± 3.09 ^c–h^	23.37 ± 4.93 ^a–i^
Group 4 PLA/G-EOs + AgNPs	6	30.86 ± 3.69 ^a–d^	25.49 ± 1.83 ^a–e^	18.99 ± 3.15 ^ab^
	9	33.83 ± 3.10 ^b–h^	27.47 ± 1.80 ^b–h^	22.72 ± 5.06 ^a–h^
	12	33.64 ± 3.38 ^b–h^	26.02 ± 2.61 ^a–g^	21.78 ± 5.24 ^a–g^
	18	32.08 ± 2.99 ^a–f^	26.22 ± 2.20 ^a–g^	22.44 ± 4.19 ^a–h^
Irradiated	0	32.21 ± 3.65 ^a–g^	27.18 ± 3.39 ^a–h^	24.10 ± 5.77 ^b–i^
Commercial control + irradiation (I)	6	31.77 ± 2.81 ^a–e^	27.97 ± 1.91 ^c–h^	22.23 ± 4.35 ^a–g^
	9	35.78 ± 2.31 ^f–h^	27.18 ± 2.21 ^a–h^	24.99 ± 3.20 ^d–i^
	12	33.21 ± 3.66 ^b–h^	25.76 ± 4.11 ^a–f^	22.82 ± 6.01 ^a–h^
Group 1 PLA/G + I	6	32.94 ± 1.91 ^b–h^	27.67 ± 3.07 ^b–h^	22.92 ± 3.66 ^a–i^
	9	34.07 ± 2.48 ^b–h^	26.97 ± 2.85 ^a–g^	23.06 ± 4.75 ^a–i^
	12	32.96 ± 2.24 ^b–h^	23.82 ± 2.79 ^a^	19.53 ± 4.05 ^a–d^
	18	30.40 ± 2.02 ^ab^	25.40 ± 3.44 ^a–d^	19.11 ± 4.09 ^a–c^
Group 2 PLA/G-AgNPs + I	6	34.72 ± 3.49 ^c–h^	30.30 ± 1.50 ^h^	27.89 ± 4.56 ^h–i^
	9	34.24 ± 2.96 ^b–h^	27.04 ± 3.48 ^a–h^	23.48 ± 5.37 ^a–i^
	12	34.46 ± 3.43 ^c–h^	27.42 ± 3.77 ^b–h^	24.20 ± 6.43 ^b–i^
	18	31.91 ± 3.31 ^a–f^	25.79 ± 4.61 ^a–g^	21.39 ± 6.27 ^a–f^
	24	31.86 ± 1.72 ^a–f^	25.66 ± 1.85 ^ae^	22.37 ± 3.31 ^a–h^
Group 3 PLA/G-EOs + I	6	36.52 ± 3.51 ^h^	28.44 ± 2.92 ^d–h^	28.39 ± 5.24 ^i^
	9	36.29 ± 2.12 ^h^	28.84 ± 2.11 ^e–h^	25.59 ± 4.12 ^e–i^
	12	33.53 ± 3.35 ^b–h^	26.99 ± 1.43 ^a–g^	22.18 ± 3.90 ^a–g^
	18	31.88 ± 2.04 ^a–f^	25.49 ± 1.75 ^a–e^	19.62 ± 3.61 ^a–d^
	24	31.83 ± 3.47 ^a–f^	25.18 ± 5.07 ^a–d^	21.70 ± 6.38 ^a–g^
Group 4 PLA/G-EOs + AgNPs + I	6	32.33 ± 3.06 ^a–g^	26.41 ± 3.01 ^a–g^	23.81 ± 3.77 ^a–i^
	9	30.78 ± 6.41 ^a–d^	27.46 ± 2.87 ^b–h^	22.73 ± 5.31 ^a–h^
	12	34.19 ± 2.57 ^b–h^	28.01 ± 2.59 ^c–h^	24.71 ± 4.77 ^c–i^
	18	30.56 ± 1.50 ^a–c^	24.30 ± 2.07 ^ab^	18.74 ± 2.59 ^ab^
	24	29.02 ± 5.33 ^a^	23.88 ± 2.96 ^a^	18.33 ± 3.02 ^a^

In each column, means ± standard deviation followed by the same letter are not significantly different based on Duncan’s multiple range test (*p* > 0.05).

**Table 5:** Effect of packaging and irradiation treatment on the firmness, total soluble solids (TSS), total phenolic content (TPC), and anthocyanin content of strawberries during storage.

Treatment	Day	Firmness (N)	TSS (°Brix)	TPC (mg GAE/100 g)	Anthocyanin Content (mg CGE/L)
Non-irradiated	0	0.78 ± 0.02 ^q^	8.07 ± 0.12 ^ef^	202.68 ± 0.65 ^t^	103.31 ± 0.17 ^c^
Commercial control	6	0.50 ± 0.04 ^c–h^	8.07 ± 0.10 ^ef^	195.24 ± 0.05 ^r^	118.30 ± 0.08 ^n^
	9	0.47 ± 0.08 ^b–g^	9.00 ± 0.20 ^g^	204.78 ± 0.02 ^u^	154.38 ± 0.11 ^hh^
	12	0.46 ± 0.10 ^b–f^	7.07 ± 0.12 ^a^	180.88 ± 0.07 ^j^	121.89 ± 0.01 ^q^
Group 1 PLA/G	6	0.53 ± 0.06 ^f–k^	8.07 ± 0.14 ^ef^	224.58 ± 0.05 ^y^	102.13 ± 0.03 ^b^
	9	0.46 ± 0.04 ^b–f^	8.33 ± 0.12 ^f^	181.92 ± 0.07 ^k^	141.44 ± 0.14 ^cc^
	12	0.45 ± 0.13 ^b–f^	8.33 ± 0.12 ^f^	161.16 ± 0.13 ^d^	154.69 ± 0.10 ^ii^
Group 2 PLA/G-AgNPs	6	0.51 ± 0.09 ^c–i^	8.00 ± 0.20 ^ef^	194.35 ± 3.26 ^q^	110.95 ± 0.09 ^h^
	9	0.49 ± 0.07 ^c–g^	7.93 ± 0.12 ^def^	208.16 ± 0.01 ^v^	126.92 ± 0.18 ^t^
	12	0.49 ± 0.07 ^c–g^	7.93 ± 0.12 ^def^	165.50 ± 0.06 ^f^	117.20 ± 0.05 ^l^
	18	0.44 ± 0.08 ^b–e^	7.40 ± 0.20 ^ab^	163.40 ± 0.13 ^e^	134.77 ± 0.13 ^y^
Group 3 PLA/G-EOs	6	0.48 ± 0.05 ^b–g^	7.93 ± 0.31 ^def^	185.98 ± 0.01 ^m^	120.31 ± 0.07 ^p^
	9	0.48 ± 0.03 ^b–g^	8.00 ± 0.40 ^ef^	184.44 ± 0.03 ^l^	145.76 ± 0.09 ^ee^
	12	0.46 ± 0.04 ^b–f^	8.07 ± 0.21 ^ef^	163.32 ± 0.23 ^e^	140.17 ± 0.14 ^bb^
	18	0.44 ± 0.06 ^bcd^	7.53 ± 0.12 ^bc^	179.63 ± 0.06 ^i^	121.94 ± 0.15 ^q^
Group 4 PLA/G-EOs + AgNPs	6	0.62 ± 0.06 ^mno^	8.00 ± 0.40 ^ef^	213.07 ± 0.02 ^w^	103.34 ± 0.05 ^c^
	9	0.58 ± 0.03 ^i–n^	8.13 ± 0.42 ^ef^	204.86 ± 0.02 ^u^	153.92 ± 0.18 ^gg^
	12	0.52 ± 0.11 ^f–k^	8.20 ± 0.40 ^ef^	154.64 ± 0.03 ^b^	106.55 ± 0.05 ^e^
	18	0.44 ± 0.09 ^bcd^	7.07 ± 0.12 ^a^	163.67 ± 0.16 ^e^	109.96 ± 0.09 ^g^
Irradiated	0	0.79 ± 0.03 ^q^	8.13 ± 0.12 ^ef^	208.12 ± 0.01 ^v^	109.10 ± 0.08 ^f^
Commercial control + irradiation (I)	6	0.54 ± 0.05 ^g–l^	8.33 ± 0.12 ^f^	212.60 ± 0.03 ^w^	131.38 ± 0.2 ^u^
	9	0.48 ± 0.06 ^c–g^	8.33 ± 0.31 ^f^	201.08 ± 0.04 ^s^	173.54 ± 0.13 ^ll^
	12	0.45 ± 0.07 ^b–e^	9.00 ± 0.20 ^g^	165.13 ± 0.03 ^f^	117.90 ± 0.03 ^m^
Group 1 PLA/G + I	6	0.60 ± 0.10 ^k–o^	8.07 ± 0.12 ^ef^	244.27 ± 0.12 ^aa^	150.34 ± 0.26 ^ff^
	9	0.55 ± 0.10 ^g–m^	8.00 ± 0.20 ^ef^	192.64 ± 0.01 ^p^	169.13 ± 0.16 ^kk^
	12	0.49 ± 0.04 ^c–g^	8.07 ± 0.12 ^ef^	165.22 ± 0.06 ^f^	131.42 ± 0.10 ^u^
	18	0.45 ± 0.07 ^b–e^	7.07 ± 0.12 ^a^	164.84 ± 0.18 ^f^	103.79 ± 0.05 ^d^
Group 2 PLA/G-AgNPs + I	6	0.66 ± 0.06 ^op^	8.07 ± 0.10 ^ef^	209.01 ± 0.04 ^v^	114.79 ± 0.06 ^k^
	9	0.61 ± 0.10 ^l–o^	8.07 ± 0.12 ^ef^	192.76 ± 0.01 ^p^	142.56 ± 0.11 ^dd^
	12	0.60 ± 0.05 ^l–o^	8.00 ± 0.00 ^ef^	179.18 ± 0.05 ^i^	135.45 ± 0.08 ^z^
	18	0.52 ± 0.04 ^d–j^	8.07 ± 0.20 ^ef^	190.78 ± 0.05 ^o^	132.52 ± 0.04 ^w^
	24	0.43 ± 0.09 ^bc^	8.00 ± 0.40 ^ef^	192.10 ± 0.59 ^p^	155.26 ± 0.16 ^jj^
Group 3 PLA/G-EOs + I	6	0.70 ± 0.08 ^p^	8.07 ± 0.12 ^ef^	187.54 ± 0.05 ^n^	98.65 ± 0.10 ^a^
	9	0.63 ± 0.07 ^no^	8.13 ± 0.12 ^ef^	221.02 ± 0.06 ^x^	137.32 ± 0.22 ^aa^
	12	0.59 ± 0.07 ^j–o^	8.00 ± 0.20 ^ef^	190.48 ± 0.04 ^o^	134.46 ± 0.74 ^x^
	18	0.52 ± 0.07 ^e–j^	8.07 ± 0.23 ^ef^	156.96 ± 0.15 ^c^	122.82 ± 0.03 ^s^
	24	0.36 ± 0.14 ^a^	8.07 ± 0.12 ^ef^	148.09 ± 0.16 ^a^	132.14 ± 0.04 ^v^
Group 4 PLA/G-EOs + AgNPs + I	6	0.66 ± 0.08 ^op^	8.20 ± 0.20 ^ef^	227.87 ± 0.06 ^z^	118.77 ± 0.15 ^o^
	9	0.62 ± 0.05 ^mno^	8.13 ± 0.23 ^ef^	221.63 ± 0.02 ^x^	122.56 ± 0.02 ^r^
	12	0.57 ± 0.07 ^h–n^	7.80 ± 0.20 ^cde^	180.76 ± 0.05 ^j^	102.10 ± 0.03 ^b^
	18	0.53 ± 0.07 ^f–k^	7.87 ± 0.23 ^cde^	176.19 ± 0.11 ^h^	113.38 ± 0.18 ^i^
	24	0.40 ± 0.09 ^ab^	7.60 ± 0.20 ^bcd^	167.14 ± 0.14 ^g^	113.81 ± 0.12 ^j^

In each column, means ± standard deviation followed by the same letter are not significantly different based on Duncan’s multiple range test (*p* > 0.05). Letters evolve in alphabetical order in single letters (a to z for position 1 to 26) followed by double letters (aa, bb, etc. for positions > 26). TSS: total soluble solids; TPC: total phenolic compounds.

**Table 6:** Effect of packaging and irradiation treatment on the total ascorbic acid, ascorbic acid, and dehydroascorbic acid content of strawberries during storage.

Treatment	Day	Total Ascorbic Acid (mg/100 g)	Ascorbic Acid (mg/100 g)	Dehydroascorbic Acid (mg/100 g)
Non-irradiated	0	84.34 ± 1.19 ^p^	50.91 ± 0.45 ^o^	33.43 ± 0.78 ^o^
Commercial control	6	61.55 ± 0.61 ^ij^	36.64 ± 0.46 ^cd^	24.91 ± 1.07 ^lm^
	12	43.75 ± 0.42 ^b^	31.68 ± 0.28 ^b^	12.07 ± 0.58 ^bc^
Group 1 PLA/G	6	63.92 ± 1.22 ^k^	46.34 ± 0.34 ^lm^	17.58 ± 0.88 ^fg^
	12	59.68 ± 0.45 ^g–j^	44.81 ± 0.59 ^klm^	14.88 ± 1.01 ^de^
Group 2 PLA/G-AgNPs	6	72.23 ± 0.15 ^n^	46.67 ± 0.61 ^mn^	25.56 ± 0.46 ^m^
	12	58.78 ± 0.27 ^gh^	44.39 ± 0.36 ^jkl^	14.39 ± 0.31 ^de^
	18	46.86 ± 0.35 ^c^	39.62 ± 0.37 ^ef^	7.24 ± 0.62 ^a^
Group 3 PLA/G-EOs	6	61.66 ± 0.15 ^j^	40.84 ± 1.83 ^fgh^	20.82 ± 1.68 ^h–k^
	12	60.98 ± 0.82 ^ij^	40.63 ± 0.09 ^fg^	20.35 ± 0.90 ^hij^
	18	49.79 ± 1.15 ^d^	35.23 ± 0.38 ^c^	14.56 ± 1.53 ^de^
Group 4 PLA/G-EOs + AgNPs	6	70.61 ± 0.61 ^mn^	48.50 ± 0.15 ^n^	22.11 ± 0.76 ^jk^
	12	61.72 ± 0.18 ^j^	44.34 ± 0.86 ^jkl^	17.38 ± 1.04 ^fg^
	18	57.68 ± 1.30 ^fg^	42.38 ± 0.94 ^g–j^	15.25 ± 0.53 ^de^
Irradiated	0	79.89 ± 1.71 ^o^	44.70 ± 2.09 ^klm^	35.19 ± 1.62 ^o^
Commercial control + irradiation (I)	6	56.52 ± 0.33 ^f^	30.42 ± 0.08 ^b^	26.10 ± 0.41 ^m^
	12	40.99 ± 0.14 ^a^	27.80 ± 0.09 ^a^	13.18 ± 0.16 ^cd^
Group 1 PLA/G film + I	6	68.02 ± 0.92 ^l^	41.81 ± 0.15 ^f–i^	26.21 ± 1.07 ^m^
	12	59.44 ± 1.45 ^ghi^	37.55 ± 2.29 ^d^	21.89 ± 1.93 ^ijk^
	18	52.75 ± 2.54 ^e^	36.47 ± 0.62 ^cd^	16.28 ± 1.91 ^ef^
Group 2 PLA/G-AgNPs + I	6	68.78 ± 0.76 ^lm^	41.84 ± 1.11 ^f–i^	26.94 ± 0.35 ^m^
	12	60.14 ± 0.74 ^hij^	40.36 ± 1.36 ^efg^	19.78 ± 2.10 ^hi^
	18	53.02 ± 0.30 ^e^	38.41 ± 1.25 ^de^	14.61 ± 1.46 ^de^
Group 3 PLA/G-EOs + I	6	70.29 ± 1.07 ^mn^	43.90 ± 0.69 ^ijk^	26.39 ± 0.37 ^m^
	12	64.45 ± 0.82 ^k^	41.48 ± 0.08 ^fgh^	22.97 ± 0.84 ^kl^
	18	50.83 ± 0.56 ^d^	39.67 ± 0.14 ^ef^	11.16 ± 0.54 ^bc^
Group 4 PLA/G-EOs + AgNPs + I	6	72.34 ± 0.00 ^n^	42.92 ± 0.14 ^h-k^	29.42 ± 0.14 ^n^
	12	60.40 ± 0.55 ^hij^	41.57 ± 0.23 ^fgh^	18.83 ± 0.77 ^gh^
	18	50.24 ± 0.28 ^d^	39.95 ± 0.43 ^ef^	10.30 ± 0.71 ^b^

In each column, means ± standard deviation followed by the same letter are not significantly different based on Duncan’s multiple range test (*p* > 0.05).

## Data Availability

The original contributions presented in this study are included in the article. Further inquiries can be directed to the corresponding author.

## Supplementary Materials

The following supporting information can be downloaded at: https://www.mdpi.com/article/10.3390/foods15162836/s1, Figure S1: Physical appearance of the PLA/G-based bioactive films (a: PLA/G, b: PLA/G- AgNPs, c: PLA/G-EOs, d: PLA/G-EOs + AgNPs); Figure S2: DSC curves of the PLA/G active packaging films at the first heating cycle.
