# Supplementary material for: Effects of PLA-Based Antimicrobial Composite Films and Low-Energy X-Ray Irradiation on the Quality and Shelf Life of Strawberries
Source: Foods. 2026 Aug 14;15(16):2836. doi: 10.3390/foods15162836 (PMC13512031; doi:10.3390/foods15162836)
Supplement: Supplementary file 1 [file foods-15-02836-s001.zip › foods-4380110-supplementary.pdf]

Supplementary material:

Figure **S1:**

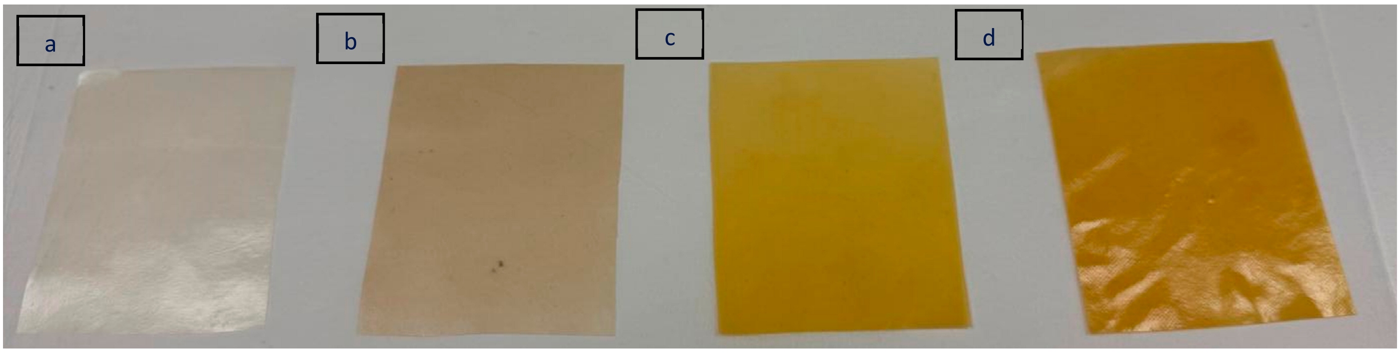

Figure S1. Physical appearance of the PLA/G-based bioactive films (a: PLA/G, b: PLA/G- AgNPs, c: PLA/G-EOs, d: PLA/G-EOs + AgNPs).

Figure **S2:**

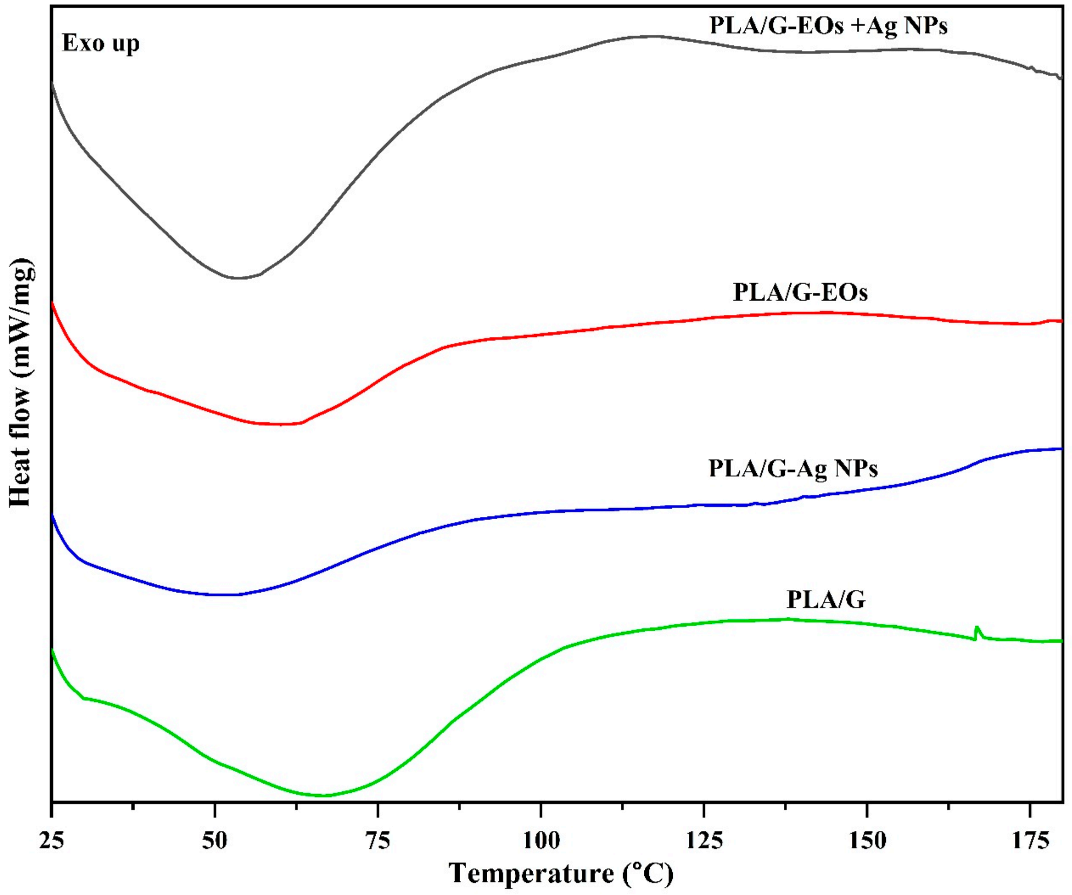

Figure S2. DSC curves of the PLA/G active packaging films at the first heating cycle.
